# Phosphatidic Acid Produced by Phospholipase D Promotes RNA Replication of a Plant RNA Virus

**DOI:** 10.1371/journal.ppat.1004909

**Published:** 2015-05-28

**Authors:** Kiwamu Hyodo, Takako Taniguchi, Yuki Manabe, Masanori Kaido, Kazuyuki Mise, Tatsuya Sugawara, Hisaaki Taniguchi, Tetsuro Okuno

**Affiliations:** 1 Laboratory of Plant Pathology, Graduate School of Agriculture, Kyoto University, Kyoto, Japan; 2 Institute for Enzyme Research, University of Tokushima, Tokushima, Japan; 3 Laboratory of Marine Bioproducts Technology, Graduate School of Agriculture, Kyoto University, Kyoto, Japan; Agriculture and Agri-Food Canada, CANADA

## Abstract

Eukaryotic positive-strand RNA [(+)RNA] viruses are intracellular obligate parasites replicate using the membrane-bound replicase complexes that contain multiple viral and host components. To replicate, (+)RNA viruses exploit host resources and modify host metabolism and membrane organization. Phospholipase D (PLD) is a phosphatidylcholine- and phosphatidylethanolamine-hydrolyzing enzyme that catalyzes the production of phosphatidic acid (PA), a lipid second messenger that modulates diverse intracellular signaling in various organisms. PA is normally present in small amounts (less than 1% of total phospholipids), but rapidly and transiently accumulates in lipid bilayers in response to different environmental cues such as biotic and abiotic stresses in plants. However, the precise functions of PLD and PA remain unknown. Here, we report the roles of PLD and PA in genomic RNA replication of a plant (+)RNA virus, *Red clover necrotic mosaic virus* (RCNMV). We found that RCNMV RNA replication complexes formed in *Nicotiana benthamiana* contained PLDα and PLDβ. Gene-silencing and pharmacological inhibition approaches showed that PLDs and PLDs-derived PA are required for viral RNA replication. Consistent with this, exogenous application of PA enhanced viral RNA replication in plant cells and plant-derived cell-free extracts. We also found that a viral auxiliary replication protein bound to PA *in vitro*, and that the amount of PA increased in RCNMV-infected plant leaves. Together, our findings suggest that RCNMV hijacks host PA-producing enzymes to replicate.

## Author Summary

All characterized eukaryotic positive-strand RNA [(+)RNA] viruses replicate their genomes using the viral replication complexes (VRCs), which contain multiple viral and host components, on intracellular membranes. Phospholipids are major constituents of cellular membranes; however, the function(s) of phospholipids in genome replication of (+)RNA viruses remains largely unknown. Here, we show that *Red clover necrotic mosaic virus* (RCNMV), a plant (+)RNA virus, induces a high accumulation of phosphatidic acid (PA) in infected plant leaves. PA-producing enzymes, phospholipase Dα (PLDα) and PLDβ, are associated with RCNMV VRCs. PA interacts with the viral replication protein and enhances the viral replication by upregulating the activity/assembly of the VRCs *in vitro*. In summary, RCNMV alters cellular lipid metabolism via PLD to establish a suitable environment for viral replication.

## Introduction

Positive-strand RNA [(+)RNA] viruses are the most abundant plant viruses, and include many viruses economically important in agriculture. (+)RNA plant viruses have a limited coding capacity. To replicate and achieve successful infection in their hosts, they need to use host proteins, membranes, lipids, and metabolites. All characterized eukaryotic (+)RNA viruses replicate their genomes using viral replication complexes (VRCs), which contain multiple viral and host components on intracellular membranes [1–6]. A growing number of studies have suggested that plant viruses have evolved ways to hijack plant host factors and reprogram host cell metabolism for their successful infection [6, 7]. Conversely, plants have evolved the ability to recognize viruses through specific interaction with viral proteins or viral double-stranded RNA intermediates for restricting virus infection [8, 9]. Viruses must circumvent or suppress such surveillance systems and host defense mechanisms. Thus, viruses must be evolved to achieve a good balance between hijacking/reprogramming host factors for efficient viral replication and avoiding the danger of stimulating antiviral defense responses.

*Red clover necrotic mosaic virus* (RCNMV) is a (+)RNA plant virus and a member of the genus *Dianthovirus* in the family *Tombusviridae*. The genome of RCNMV consists of RNA1 and RNA2. RNA1 encodes a p27 auxiliary replication protein, p88^pol^ RNA-dependent RNA polymerase (RdRp), and a coat protein [10]. RNA2 encodes a movement protein that is required for viral cell-to-cell movement [10, 11]. p27, p88^pol^, and host proteins form a 480-kDa replicase complex, which is a key player in the viral RNA replication [12]. p27 and p88^pol^ colocalize at the endoplasmic reticulum (ER) [13, 14], where RCNMV replication takes place [15]. Our previous studies showed that RCNMV uses host heat shock proteins (HSPs), HSP70 and HSP90 [16], and ADP-ribosylation factor 1 (Arf1) [15] for the formation of the 480-kDa replicase complex and p27-induced ER membrane alterations. Arf1 is a small GTPase that regulates COPI vesicle formation. Sar1, another small GTPase that regulates COPII vesicle-mediated trafficking, and Arf1 are recruited from their original subcellular locations to RCNMV replication sites via p27, and p27 interferes with host membrane trafficking pathway in plant cells [15, 17]. Mammalian and yeast Arf1 recruits and/or stimulates its effector proteins, including a coatomer, phosphatidylinositol 4 kinase III β (PI4KIIIβ), and phospholipase D (PLD) [18]. Arf1 can activate mammalian PLD1 and PLD2 directly. PLD hydrolyses structural phospholipids such as phosphatidylcholine (PC) and phosphatidylethanolamine (PE) to produce phosphatidic acid (PA) and remaining headgroups. PA production resulting from Arf1-mediated PLD activation has been proposed to be associated with vesicle formation [19].

The 12 different PLD isoforms encoded in the *Arabidopsis thaliana* genome are classified into six groups (α, β, γ, δ, ε, and ζ) based on sequence similarity and *in vitro* activity [20]. PLDζ1 and ζ2 have N-terminal phox homology (PX) and pleckstrin homology (PH) domains and share high sequence similarities to two PX/PH-PLDs in mammals. The remaining PLDs contain the Ca^2+^-dependent phospholipid-binding C2 domain and are unique to plants.

PA is normally present in small amounts (less than 1% of total phospholipids), but rapidly and transiently accumulates in lipid bilayers in response to different abiotic stresses such as dehydration, salt, and osmotic stress [20–22]. PA also accumulates in response to several microbe-associated molecular patterns (MAMPs) in plant cells and positively regulates salicylic acid (SA)-mediated defense signaling [23–27]. Moreover, effector proteins of bacterial and fungal pathogens, such as *Cladosporium fulvum* Avr4 and *Pseudomonas syringae* AvrRpm1 and AvrRpt2, trigger PA accumulation in their host cells, and multiple PLD isoforms contribute to AvrRpm1-triggered resistance in *Arabidopsis thaliana* [28–30]. PLDδ plays a positive role in MAMPs-triggered cell wall based immunity and nonhost resistance against *Blumeria graminis* f. sp. *hordei* [31]. Moreover, overexpression of rice diacylglycerol (DAG) kinase, which catalyzes the conversion of DAG to PA, enhances resistance against *tobacco mosaic virus* and *Phytophthora parasitica* infections in tobacco plants [32]. In accordance with this, direct application of PA to leaves has been shown to induce the expression of *pathogenesis-related* (*PR*) genes and cell death [28,33]. These findings indicate that PA is a positive regulator in plant defense against pathogens. In contrast, PLDβ1 acts like a negative regulator of the generation of reactive oxygen species (ROS), the expression of *PR* genes, and plant defenses against biotrophic pathogens in rice and *Arabidopsis* [34–36].

In this study, using two-step affinity purification and liquid chromatography–tandem mass spectrometry (LC/MS/MS) analysis, we identified *Nicotiana benthamiana* PLDα and PLDβ as interaction partners of RCNMV replication protein, p88^pol^. Gene-silencing and pharmacological inhibition approaches show that PLDs-derived PA plays a positive role in viral RNA replication. Consistent with this role, direct application of PA to plant cells or plant-derived cell-free extracts enhanced RCNMV RNA replication and negative-strand RNA synthesis, respectively. We found that p27 auxiliary replication protein interacted with PA *in vitro* and that the accumulation of PA increased in RCNMV-infected plant leaves. Together, our findings suggest that RCNMV hijacks host PA-producing enzymes to achieve successful RNA replication.

## Results

To identify putative host proteins associated with the RCNMV replicase complex, we expressed six-His/FLAG-tagged p27 and p88^pol^ replication proteins (p27-HF and p88^pol^-HF, respectively) together with an RNA2 replication template via agroinfiltration in *N*. *benthamiana*. Two-days after infiltration (dai), RCNMV replication proteins were purified via sequential affinity purification using nickel-agarose beads and FLAG-affinity resins as described in the Materials and Methods section. Note that p27-HF and p88^pol^-HF replication proteins can support RNA2 replication in *N*. *benthamiana* plants, although their activities were low compared with those of non-tagged p27 and p88 ^pol^ (S1 Fig).

**Fig 1:**
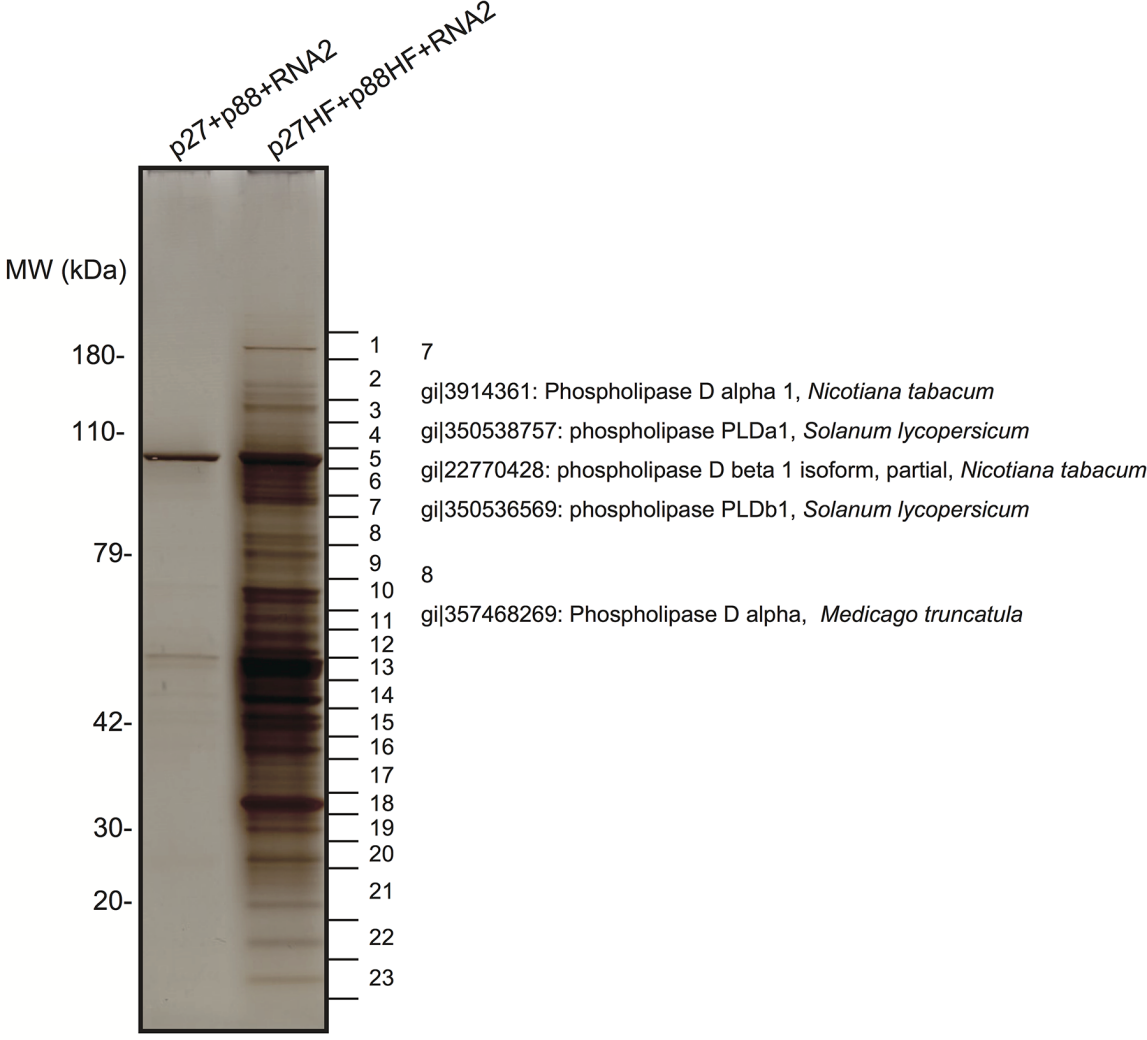
Identification of proteins copurified with RCNMV replication proteins. The solubilized total fractions prepared from *Agrobacterium*-infiltrated leaves expressing p27 plus p88 plus RNA2 or p27-His-FLAG (p27-HF) plus p88-His-FLAG (p88-HF) plus RNA2 were subjected to affinity purification with Ni-NTA agarose beads, followed by anti-FLAG affinity resins. The affinity-purified fractions were subjected to SDS-PAGE and stained using MS-compatible silver staining. Protein bands were excised, subjected to in-gel digestion, and analyzed by tandem mass spectrometry. Some of the proteins that were identified in LC/MS/MS analysis are listed in S1 Table.

The purified fraction was subjected to sodium dodecyl sulfate-polyacrylamide gel electrophoresis to separate the proteins that copurified with RCNMV replication proteins. A silver-stained gel showed the presence of many protein bands that were absent in the control fraction prepared from *Agrobacterium*-infiltrated *N*. *benthamiana* leaves expressing non-tagged p27 and p88^pol^ together with RNA2 (Fig 1). LC/MS/MS analysis of the isolated proteins excised from gels led to the identification of many host proteins (Fig 1 and S1 Table). These proteins included PLDα and PLDβ, and several Arf1 effector proteins, such as coatomer subunits and clathrin heavy chain, in addition to previously identified host factors, HSP70 and HSP90 [16], and Arf1 [15]. It is known that the activities of yeast and mammalian PLDs are stimulated by Arf1 [37] and Arf1 is an essential host factor in RCNMV RNA replication [15]. Therefore, we investigated whether PLDα and PLDβ are also required for RCNMV RNA replication.

**Fig 2:**
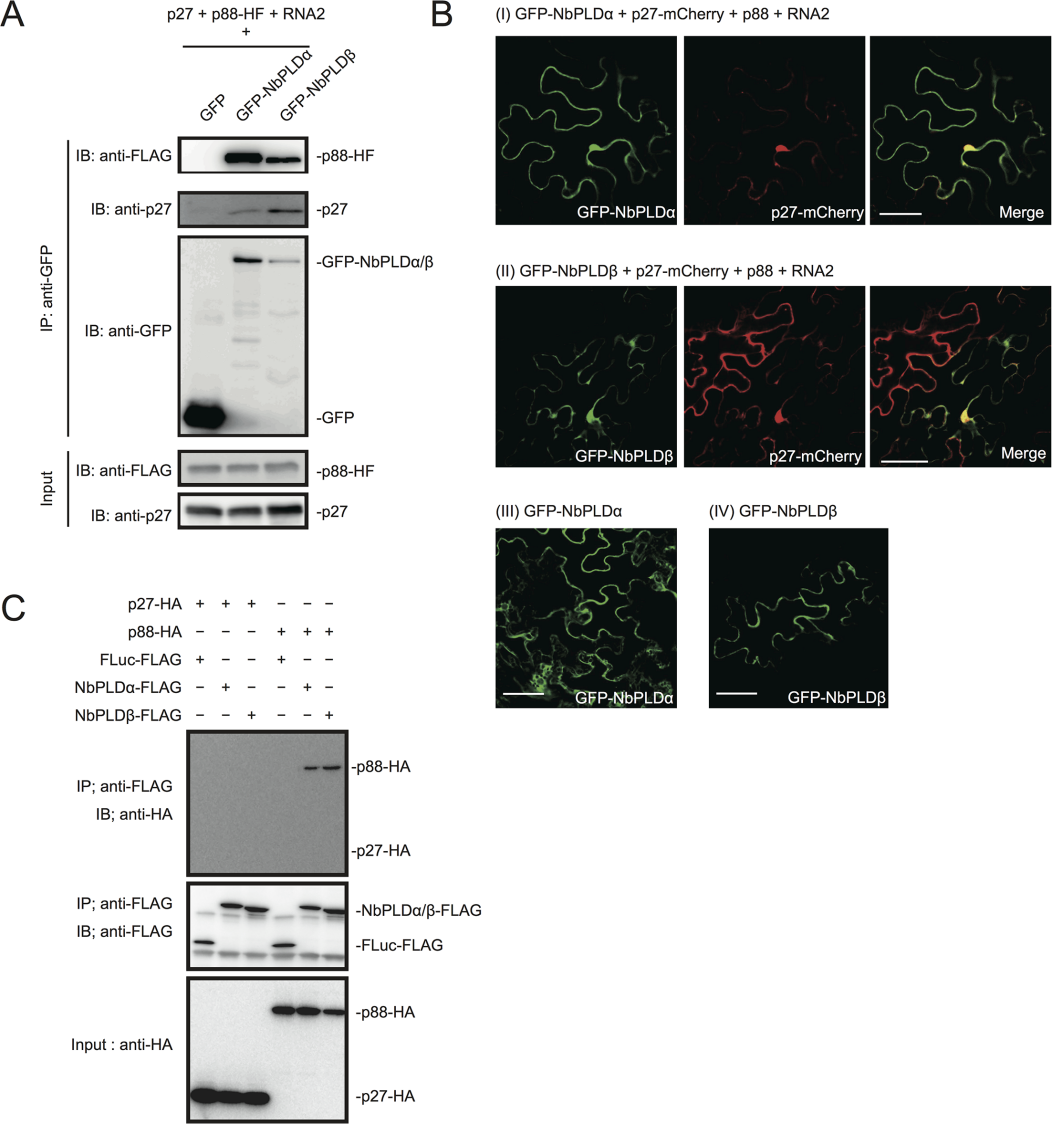
RCNMV replication proteins interact with NbPLDα and NbPLDβ. (A) RCNMV replication proteins interacted with NbPLDα and NbPLDβ in a co-IP assay in *N*. *benthamiana*. Green fluorescent protein (GFP)-fused NbPLDα (GFP-NbPLDα) or GFP-NbPLDβ was coexpressed with p27, six-His/FLAG-tagged p88^pol^ (p88^pol^-HF), RNA2, and p19 of RNA silencing suppressor of *Tomato bushy stunt virus* in *N*. *benthamiana* leaves. Total protein was extracted and subjected to immunoprecipitation of GFP-fused proteins by anti-GFP antibody. Proteins were analyzed by immunoblot using antibodies as indicated. (B) NbPLDα and NbPLDβ were colocalized with p27 during viral replication. GFP-NbPLDα or GFP-NbPLDβ was coexpressed with C-terminally mCherry-fused p27 (p27-mCherry), p88^pol^, RNA2, and p19 (panel I and II) or coexpressed with empty vector and p19 (panel III and IV) in *N*. *benthamiana* leaves by *Agrobacterium* infiltration. Fluorescence was visualized by confocal microscopy at 4 days after infiltration. The merging of the green and red fluorescence is shown as a yellow color. Bars, 50 μm. (C) p88^pol^ interacted with NbPLDα and NbPLDβ in a co-IP assay in BYL. Appropriate combinations of capped transcripts were added to BYL. After *in vitro* translation at 25°C for 2 hours, the extract was solubilized and subjected to immunoprecipitation of FLAG-tagged proteins by anti-FLAG antibody. Proteins were analyzed by immunoblot using antibodies as indicated.

We isolated cDNAs encoding *PLDα* and *PLDβ* from *N*. *benthamiana* leaves. Deduced amino acid sequences and peptide sequences identified from LC/MS/MS analysis are presented in S2 Fig. To confirm the association of *N*. *benthamiana* (Nb)PLDα and NbPLDβ with RCNMV replication proteins, we performed a co-immunoprecipitation (co-IP) assay in *N*. *benthamiana* plants using green fluorescent protein (GFP)-fused NbPLDα or NbPLDβ as bait proteins. We co-expressed p88^pol^-HF together with p27 and RNA2, because p88^pol^ can be detected by immunoblot in *N*. *benthamiana* only when viral RNA replication takes place [12]. Both p27 and p88^pol^-HF were co-immunoprecipitated with GFP-NbPLDα or GFP-NbPLDβ, but not with GFP (Fig 2A), confirming the association of these PLDs with RCNMV replication proteins.

To investigate whether PLDs localize at VRCs, GFP-NbPLDα or GFP-NbPLDβ was co-expressed with mCherry-fused p27 (as a marker of VRC), p88^pol^, and RNA2 via agroinfiltration in *N*. *benthamiana*. At 4 dai, the fluorescence of GFP-NbPLDα and GFP-NbPLDβ was partially merged with the fluorescence of p27-mCherry in large aggregate structures (Fig 2B panels I and II), which are induced by p27 and are thought to be the site of RCNMV RNA replication [13,15,16]. The expression of GFP-NbPLDα or GFP-NbPLDβ alone did not induce such aggregated structures (Fig 2B panels III and IV), suggesting that both NbPLDα and NbPLDβ are recruited to viral replication sites during RCNMV replication.

To investigate whether p27 or p88^pol^ interact with NbPLDα and NbPLDβ, we performed a co-IP assay in cell lysates prepared from evacuolated tobacco BY-2 protoplasts (BYL) [38]. C-terminally HA-tagged viral replication protein (p27-HA or p88^pol^-HA) was coexpressed with C-terminally FLAG-tagged PLD (NbPLDα-FLAG or NbPLDβ-FLAG) from capped transcripts in BYL. In BYL, p88^pol^-HA was easily detected by immunoblot using anti-HA antibody in the absence of other viral components after 2 hour of *in vitro* translation (Fig 2C). After *in vitro* translation, the extracts were subjected to immunoprecipitation using FLAG-affinity resin. Immunoblot analysis showed that p88^pol^-HA, but not p27-HA, was copurified with both NbPLDα-FLAG and NbPLDβ-FLAG (Fig 2C). p88^pol^-HA was not copurified with C-terminally FLAG-tagged firefly luciferase (FLuc-FLAG) that was used as a negative control, excluding the possibility that p88^pol^-HA binds to FLAG-affinity resin nonspecifically. In reciprocal co-IP experiments using HA antibody, we also found copurification of NbPLDα-FLAG with p88^pol^-HA, but not with FLuc-HA or p27-HA (S3 Fig). However, as NbPLDβ-FLAG was co-purified not only with p88^pol^-HA but also with FLuc-HA or p27-HA (S3 Fig), we could not confirm the specific interaction of p88^pol^ with NbPLDβ in BYL.

### Both PLDα and PLDβ are required for RCNMV infection

**Fig 3:**
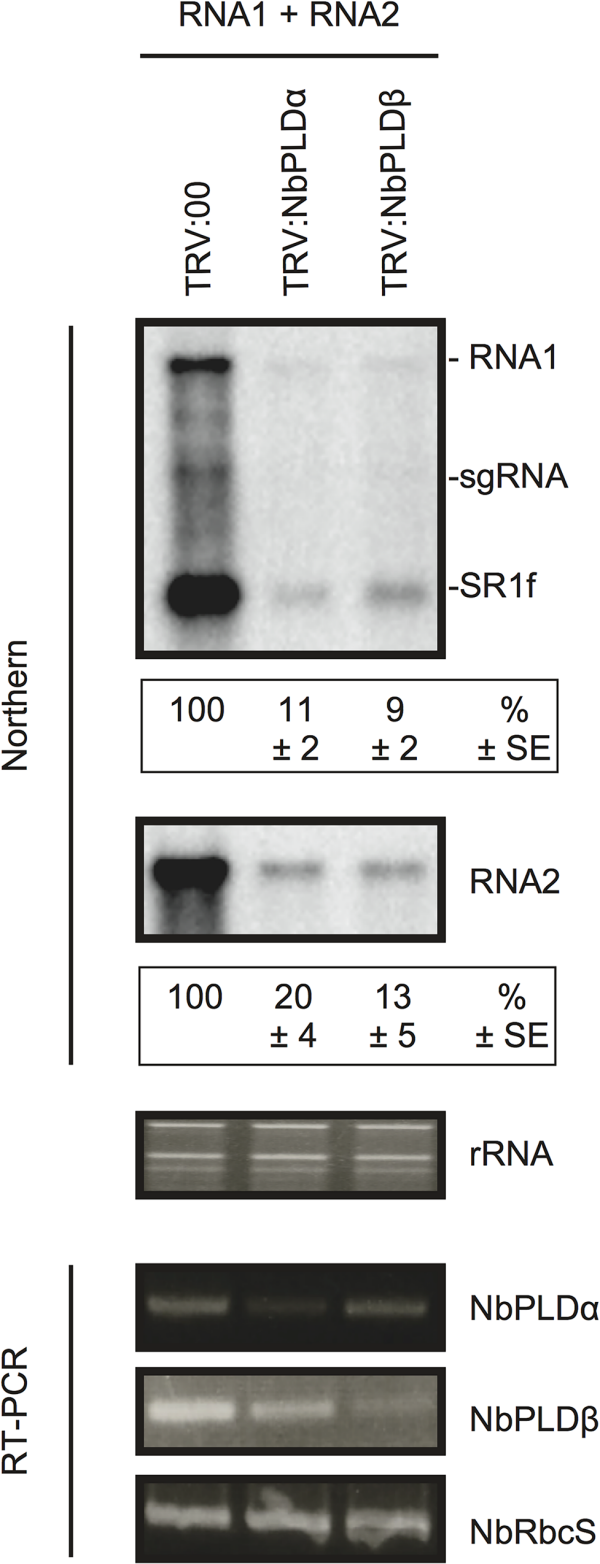
Knockdown of *NbPLDα* and *NbPLDβ* mRNAs levels via gene silencing inhibits the accumulation of RCNMV RNAs in *N*. *benthamiana*. The tobacco rattle virus (TRV) vector harboring a partial fragment of *N*. *benthamiana NbPLDα* and *NbPLDβ* mRNAs (TRV:NbPLDα and TRV:NbPLDβ, respectively) was expressed in *N*. *benthamiana* via *Agrobacterium* infiltration. The empty TRV vector (TRV:00) was used as a control. The newly developed leaves were inoculated with RCNMV RNA1 and RNA2 at 18 days after agroinfiltration. Total RNA was extracted from the mixture of three independent inoculated leaves 2 days after virus inoculation. Accumulation of RCNMV RNA was analyzed by northern blotting. Ethidium bromide-stained ribosomal RNAs (rRNAs) are shown below the northern blots, as loading controls. The numbers below the images represent the relative accumulation levels (means ± standard error) of viral RNAs (RNA1 and RNA2, respectively) using the Image Gauge program (Fuji Film), which were calculated based on three independent experiments. *NbPLDα* and *NbPLDβ* mRNA levels were assessed by RT-PCR using primers that allow the amplification of the region of coding regions that are not present in TRV vectors. RT-PCR results for the RbcS gene demonstrated that equal amounts of total RNA were used for the RT and showed equivalent efficiency of the RT reaction in the samples. sgRNA, subgenomic RNA; SR1f, a small RNA fragment that derived from non-coding region of RNA1 [47].

To test whether NbPLDα and NbPLDβ are required for infection of host plants with RCNMV, endogenous transcript levels of *NbPLDα* or *NbPLDβ* were downregulated using *Tobacco rattle virus* (TRV)-based virus-induced gene silencing (VIGS) in *N*. *benthamiana* plants. A TRV vector harboring a partial fragment of *NbPLDα* (TRV:NbPLDα) or *NbPLDβ* (TRV:NbPLDβ) was expressed via *Agrobacterium*-mediated expression. An empty TRV vector (TRV:00) was expressed as a control. Newly developed leaves were inoculated with RCNMV RNA1 and RNA2 at 18 dai. Two days after RCNMV inoculation, three inoculated leaves from three different plants were harvested and mixed, and total RNA was extracted. No morphological defects such as chlorotic and stunted phenotypes were observed at this stage (S4 Fig). Semiquantitative reverse-transcription-PCR (RT-PCR) or quantitative RT-PCR (RT-qPCR) analyses confirmed the specific reduction of *NbPLDα* or *NbPLDβ* mRNAs in plants infiltrated with TRV:NbPLDα or TRV:NbPLDβ, respectively (Figs 3 and S5). Northern blot analyses using ribonucleotide probes specifically recognizing RCNMV RNA1 or RNA2 showed that the accumulation of RCNMV RNAs was dramatically reduced in both *NbPLDα*- and *NbPLDβ*-knockdown plants compared with control plants (Fig 3).

It is known that, in PLD*β*1 knockdown transgenic rice plants, the generation of ROS and the expression of defense-related genes are induced even in the absence of pathogen infection [35]. Therefore, it is possible that the poor viral infection in *NbPLDβ-*knokedown plants was due to activated defense responses. To address this possibility, we tested the effects of *NbPLDα*- or *NbPLDβ-*knockdown by TRV-mediated VIGS on the expression of defense-related genes by RT-qPCR analysis. The defense-related genes analyzed here were SA-signaling marker genes (*PR-1*, *PR-2*, and *PR-5*) [39,40], jasmonic acid (JA)-signaling marker genes (*LOX1*, *PR-4*, and *PDF1*.*2*.) [39,41], ROS-detoxification enzymes (*APX*, *GST*, and *SOD*) [39], MAMP-triggered immunity marker genes (*CYP71D20* and *ACRE132*) [42], and mitogen-activated protein kinases (*WIPK* and *SIPK*) [42]. The expression levels of these defense-related genes were not significantly increased in *NbPLDα*- or *NbPLDβ*-knockdown plants compared with those in TRV control plants (S5 Fig), excluding the possibility that the reduced viral infection in *NbPLDα-* or *NbPLDβ-*knockdown plants are due to activated defense responses in these plants. Some genes including *PR-1*, *PR-2*, and *CYP71D20* genes were even repressed in *NbPLDα*- or *NbPLDβ*-knockdown plants (S5 Fig), consistent with the positive roles of PLDs and PLD-derived PA on plant defense signaling [24–30]. Altogether, these results suggest that both NbPLDα and NbPLDβ play a positive role in RCNMV infection.

### PA promotes viral RNA replication

**Fig 4:**
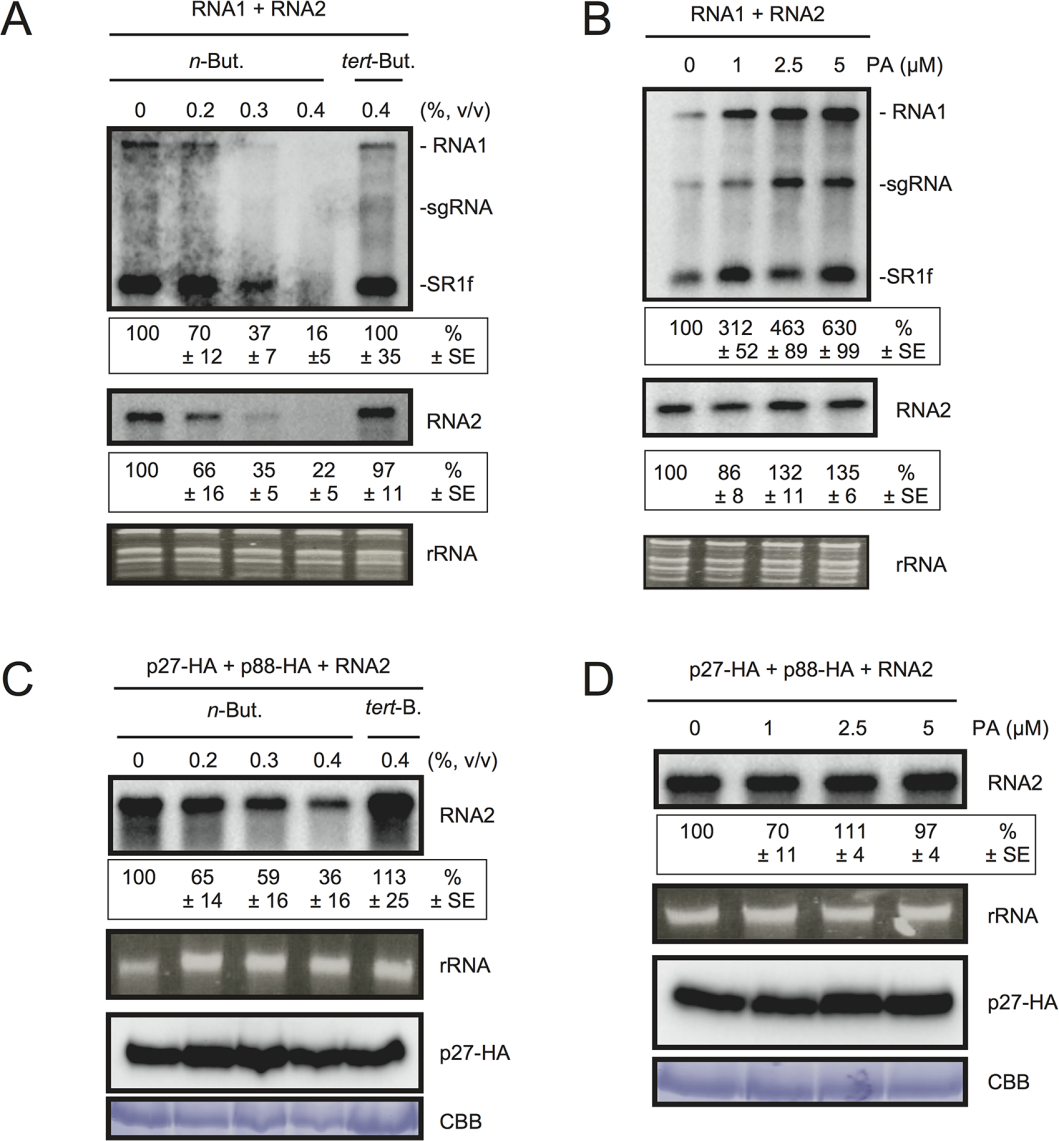
The effects of *n*-butanol and exogenously supplied PA on RCNMV RNA replication in *N*. *benthamiana* protoplasts. (A) An inhibitor of PLDs impairs RCNMV RNA replication in a single cell. *N*. *benthamiana* protoplasts were inoculated with *in vitro* transcribed RNA1 and RNA2. The inoculated protoplasts were incubated at 20°C for 18 hours in the presence of *n*-butanol. (B) An exogenously supplied phosphatidic acid (PA) enhances RCNMV RNA replication. *N*. *benthamiana* protoplasts were inoculated with *in vitro* transcribed RNA1 and RNA2. The inoculated protoplasts were incubated at 20°C for 18 hours in the presence of progressively increasing concentrations of PA. (C) An inhibitor of PLDs impairs replication of RNA2 in a single cell. *N*. *benthamiana* protoplasts were inoculated with *in vitro* transcribed RNA2 together with plasmids expressing p27-HA and p88-HA. The inoculated protoplasts were incubated at 20°C for 18 hours in the presence of *n*-butanol. (D) The effect of an exogenously supplied PA on the replication of RNA2. *N*. *benthamiana* protoplasts were inoculated with *in vitro* transcribed RNA2 together with plasmids expressing p27-HA and p88-HA. The inoculated protoplasts were incubated at 20°C for 18 hours in the presence of progressively increasing concentrations of PA. Total RNA was analyzed by northern blotting using ribonucleotide probes that recognize specifically RCNMV RNA1 and RNA2, respectively. Ethidium bromide-stained rRNAs were used as loading controls and are shown below the northern blots. The numbers below the images represent the relative accumulation levels (means ± standard error) of viral RNAs (RNA1 and RNA2, respectively) using the Image Gauge program, which were calculated based on three independent experiments. In (C) and (D), total protein was analyzed by Immunoblot using anti-HA antibody. Coomassie Brilliant Blue (CBB) staining served as a loading control. sgRNA, subgenomic RNA; SR1f, a small RNA fragment that derived from non-coding region of RNA1 [47].

To test the possible contribution of PLD-derived PA to viral RNA replication, we exploited the transphosphatidylation activity of PLDs, which uses primary alcohols as substrates to form an artificial phosphatidyl alcohol. The preferential formation of this compound impairs PA production [43]. Thus, we tested the effect of *n*-butanol that inhibits PA production by PLDs on RCNMV RNA replication. *N*. *benthamiana* protoplasts were inoculated with RCNMV RNA1 and RNA2 and incubated with *n*-butanol or *tert*-butanol, an alcohol with no inhibitory effect on PA production, and viral RNA accumulation was determined by northern blot analysis. Increasing *n*-butanol concentration caused a progressive reduction of viral RNA accumulation (Fig 4A). By contrast, viral RNA accumulation was only moderately reduced in protoplasts treated with *tert*-butanol compared with the water control. Note that *n*-butanol did not affect the accumulation of rRNA (Fig 4A). The inhibitory effect of *n*-butanol on RCNMV replication was also observed in tobacco BY-2 protoplasts (S6 Fig).

We also tested the effect of *n*-butanol and *tert*-butanol on defense-related gene expressions in *N*. *benthamiana* protoplasts. *n*-butanol did not increase the expression of defense-related genes (S7 Fig). This result corresponds well with the finding that *n*-butanol has no effects on the basal transcription of the *PR-1* gene in *Arabidopsis* seedlings [27]. In contrast, *tert*-butanol treatment caused the induction of *CYP71D20*, *ACRE132*, and *WIPK* genes (S7 Fig). This may explain weak negative effect of *tert*-butanol on RCNMV replication (Figs 4A and S6). Altogether, these results suggest that PLD-derived PA plays a positive role in viral RNA replication.

To verify further the importance of PA in viral RNA replication, commercially available soy-derived PA was supplied to RCNMV-inoculated *N*. *benthamiana* protoplasts and viral RNA accumulation was determined by northern blot analysis. Exogenously added PA enhanced the accumulation of RNA1 in a dose-dependent manner (up to 6-fold increase by 5 μM PA), whereas the effect of PA on the accumulation of RNA2 was negligible (Fig 4B). Neither exogenously supplied PC nor PE significantly affected the accumulation of viral RNA in *N*. *benthamiana* protoplasts (S8 Fig). These results suggest that PA plays an important role in RCNMV RNA replication, and that the requirement for PA may differ between RNA1 and RNA2.

Replication of RNA2 depends entirely on RNA1 simply because RNA2 uses replication proteins supplied from RNA1. The negative impact of *n*-butanol on the accumulation of RNA2 (Fig 4A) may be the indirect action of this primary alcohol through inhibition of RNA1 replication. Therefore, it is possible that RNA2 replication does not require any PLD-derived PA. To investigate this possibility, we inoculated *N*. *benthamiana* protoplasts with RNA2 and plasmids expressing p27 and p88^pol^, as suppliers of the replication proteins. The accumulation of RNA2 was decreased by *n*-butanol in a dose-dependent manner (Fig 4C). Note that in this experiment, the accumulation of p27 replication protein was not significantly changed (Fig 4C). These results indicate that PLD-derived PA was also required for the replication of RNA2 as in the case of RNA1. However, the replication of RNA2 was not enhanced by exogenously supplied PA (Fig 4D), suggesting that the threshold of PA requirement for RNA2 replication is lower than that for RNA1. The differential requirement for PA in the replication of RNA1 and RNA2 is discussed later.

**Fig 5:**
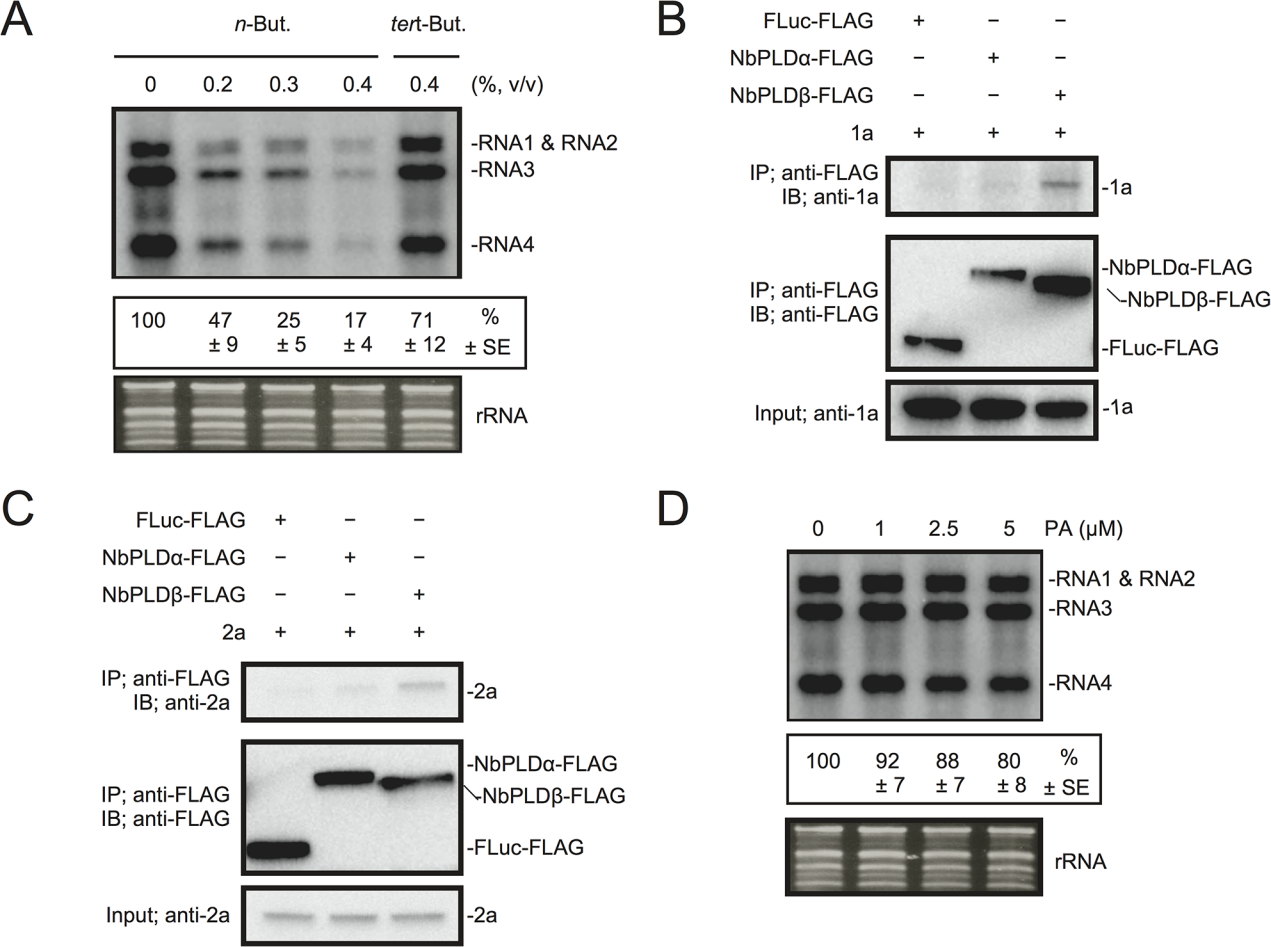
BMV RNA replication requires PLD-derived PA in *N*. *benthamiana* protoplasts. (A) An inhibitor of PLDs impairs BMV RNA replication in a single cell. *N*. *benthamiana* protoplasts were inoculated with *in vitro* transcribed BMV RNA1, RNA2, and RNA3. The inoculated protoplasts were incubated at 20°C for 18 hours in the presence of *n*-butanol. (B)-(C) Brome mosaic virus replication proteins 1a (B) and 2a^pol^ (C) interact with NbPLDβ. Appropriate combinations of capped transcripts were added to BYL. After *in vitro* translation at 25°C for 2 h, the extract was solubilized and mixed with a 10 μl bed volume of anti-FLAG M2 antibody agarose and incubated further at 4°C for 1 hours. After washing, proteins copurified with FLAG-tagged proteins were analyzed by immunoblotting with appropriate antibodies. (D) The effect of an exogenously supplied PA on the replication of BMV. *N*. *benthamiana* protoplasts were inoculated with BMV RNA1, RNA2, and RNA3 transcribed *in vitro*. The inoculated protoplasts were incubated at 20°C for 18 hours in the presence of progressively increasing concentrations of PA. Total RNA was analyzed by northern blotting using ribonucleotide probes that specifically recognize the 3’-UTR of BMV RNAs. Ethidium bromide-stained rRNA was used as a loading control and is shown below the northern blots. The numbers below the images represent the relative accumulation levels (means ± standard error) of viral RNAs (RNA1 + RNA2 + RNA3 + RNA4) using the Image Gauge program, which were calculated based on three independent experiments.

Next, we investigated the effect of *n*-butanol on RNA replication of *Brome mosaic virus* (BMV), another plant (+)RNA virus, which is unrelated to RCNMV. Increasing *n*-butanol concentration caused progressive reduction in the accumulation of BMV RNA (Fig 5A). Co-IP experiments showed interactions between BMV replication proteins and NbPLDβ-FLAG (Fig 5B and 5C). These results suggest that PLD-derived PA is also important for BMV RNA replication. However, exogenously added PA did not affect the accumulation of BMV RNA (Fig 5D) as similarly seen for RCNMV RNA2.

### PA stimulates VRC activity *in vitro*

**Fig 6:**
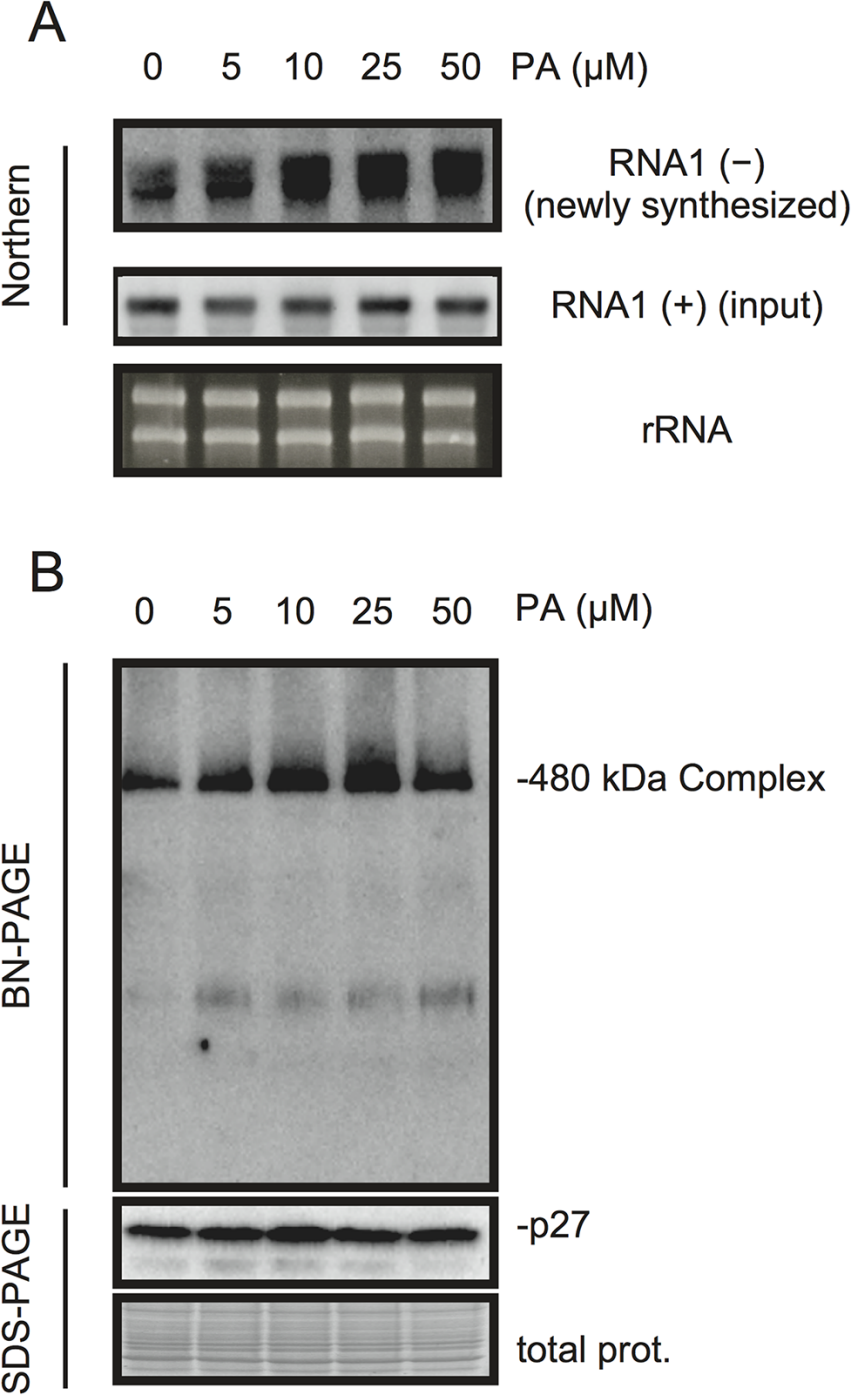
PA enhances accumulation of negative-strand RNA1 and the 480-kDa replicase complex in BYL. BYL was incubated for 4 h at 17°C with *in vitro*-transcribed RNA1 (300 ng) in the presence of various concentrations of PA as indicated. Accumulation of negative-strand RNA1 was detected by northern blotting (A). Total protein solubilized from BYL was subjected to blue native (BN)- and SDS-PAGE analyses, followed by immunoblotting with anti-p27 antisera (B). EtBr-stained rRNA and Coomassie Brilliant Blue (CBB)-stained cellular proteins are shown as loading controls.

PA acts as a second messenger in signal transduction during multiple biotic and abiotic stress responses and plays multiple roles including that for transcriptional reprogramming [20–22]. To investigate whether PA has a direct role in viral RNA replication, we took advantage of BYL, a nucleus-depleted (therefore, the effects of PA on transcriptional reprogramming are negligible) *in vitro* translation/replication system that has been used successfully to recapitulate the negative-strand RNA synthesis of RCNMV [12, 44–49]. Addition of PA into BYL stimulated the accumulation of newly synthesized negative-strand RNA (Fig 6A), and moderately enhanced the accumulation of the 480-kDa replicase complex (Fig 6B), suggesting that PA stimulates the activity and/or assembly of the viral replicase complex in a direct manner. The stimulation effects of PA on the accumulation of the 480-kDa replicase complex was less obvious than that on the accumulation of newly-synthesized viral RNA at the highest concentration of PA used in this experiment (50 μM). This result may also support a direct role for PA in the enhancement of RdRP activity rather than the formation of the replicase complex.

### p27 replication protein binds to PA *in vitro*

**Fig 7:**
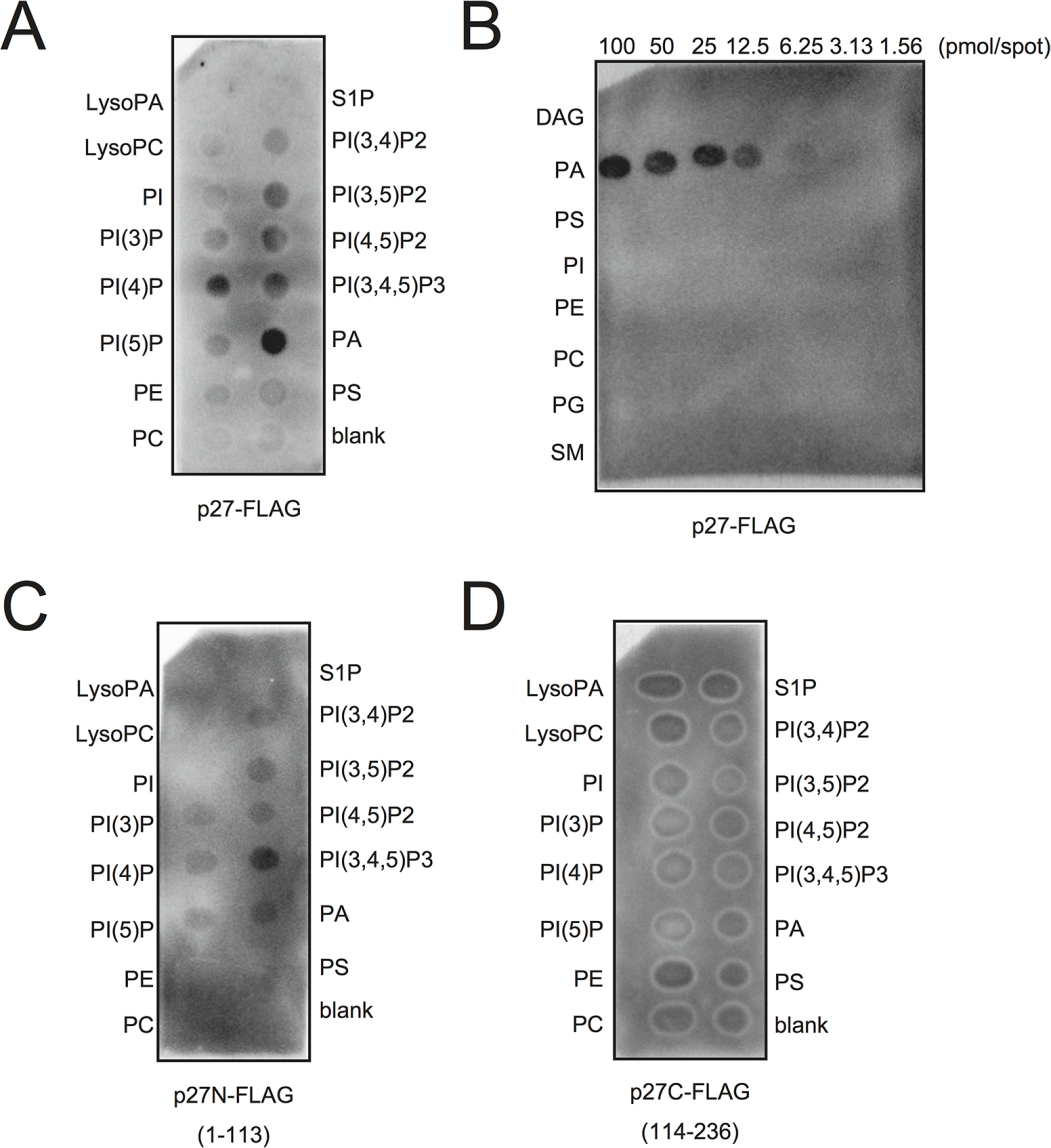
p27 interacts with PA *in vitro*. p27-FLAG or its variant proteins expressed in *E*. *coli* BL21 (DE3) were purified by Ni-NTA agarose beads. Lipid overlay analysis of various lipids with full-length p27-FLAG (A)-(B), N-terminal half of p27-FLAG (p27N-FLAG) (C), or C-terminal half of p27-FLAG (p27C-FLAG) (D). Purified protein (500 ng) was used, followed by immunoblotting with anti-FLAG antibody. LysoPA, lysophosphatidic acid; LysoPC, lysophosphochorine; PI, phosphatidylinositol; PI(3)P, phosphatidylinositol 3-phosphate; PI(4)P, phosphatidylinositol 4-phosphate; PI(5)P, phosphatidylinositol 5-phosphate; PE, phosphatidylethanolamine; PC, phosphatidylcholine; S1P, sphingosine-1-phosphate; PI(3,4)P2, phosphatidylinositol (3,4)-bisphosphate; PI(3,5)P2, phosphatidylinositol (3,5)-bisphosphate; PI(4,5)P2, phosphatidylinositol (4,5)-bisphosphate; PI(3,4,5)P3, phosphatidylinositol (3,4,5)-trisphosphate; PA, phosphatidic acid; PS, phosphatidylserine; DAG, diacylglycerol; PG, phosphatidylglycerol; SM, sphingomyelin.

Because PA directly stimulated the viral negative-strand RNA synthesis in BYL, we hypothesized that p27, the multifunctional RCNMV replication protein, has an affinity for PA. To investigate this possibility, we conducted a lipid overlay assay using bacterially expressed, purified C-terminally FLAG-tagged p27 (p27-FLAG) [49]. Purified p27-FLAG protein was incubated with phospholipid-spotted nitrocellulose membranes, and the interaction between p27-FLAG and phospholipids was detected using anti-FLAG antibody. p27 gave strong PA binding signals on the blot (Fig 7A and 7B). p27 also exhibited weak binding to phosphatidylinositol-4-phosphate (PI4P), phosphatidylinositol (3,5)-bisphosphate, phosphatidylinositol (4,5)-bisphosphate, and phosphatidylinositol (3,4,5)-trisphosphate, but negligible binding to other lipids, including PC and PE (Fig 7A and 7B). These results were consistent with the findings that neither exogenously supplied PC nor PE promoted RCNMV replication in *N*. *benthamiana* protoplasts (S8 Fig). N- or C-terminal halves of p27 fragments did not give PA binding signals (Fig 7C and 7D), suggesting that overall protein conformation may be important for p27–PA binding.

### RCNMV promotes PA accumulation *in planta*

**Fig 8:**
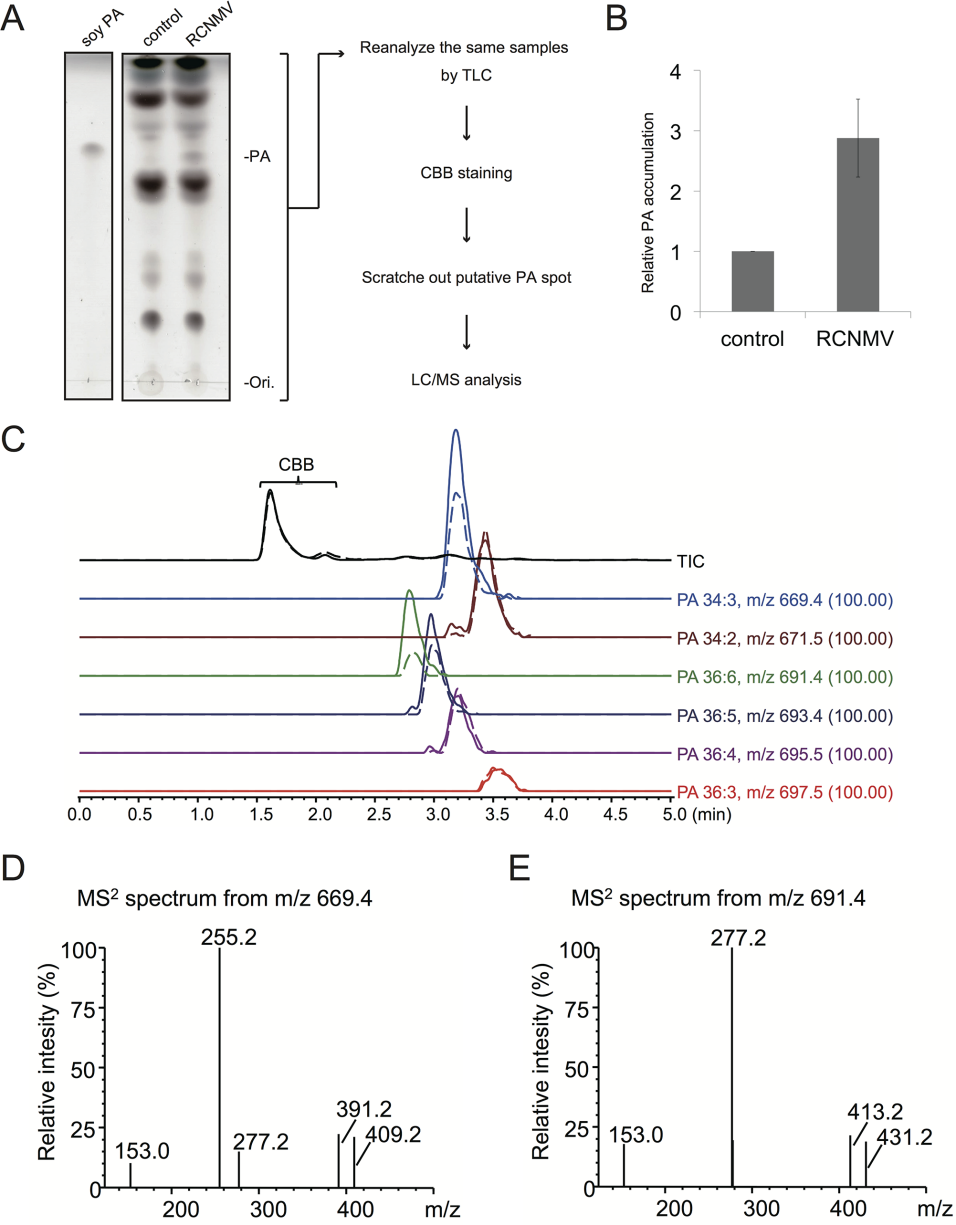
RCNMV stimulates the accumulation of PA in a plant tissue. (A) The extracted lipids from *Agrobacterium*-infiltrated leaves expressing empty vector or RCNMV RNA1 plus RNA2 were subjected to thin layer chromatography (TLC) and phospholipids were visualized by CuSO_4_ staining. The same samples were again subjected to TLC, and the spot was scratched out from Coomassie Brilliant Blue R-250 (CBB) stained TLC plates and subjected to LC/MS analysis. (B) Relative PA accumulation was quantified using ImageJ software. Bars represent means and standard error of values obtained from three independent biological samples. (C) Total ion and major extracted ion chromatograms of the TLC separated bands. The broken lines indicate the extracts from empty vector-expressed leaves, and the solid lines indicate the ones from the leaves expressing RCNMV RNA1 plus RNA2. (D)-(E), MS^2^ spectra produced by CID of m/z 669.4 (D) and m/z 691.4 (E). The signal at m/z 153.0, 255.2, and 277.2 indicate the dehydrated glycerol phosphate, carboxylate anion of C_16:0_ and C_18:3_, respectively. Ions of m/z 391.2 and 413.2 arose from the neutral loss of C_18:3,_ and m/z 409.2 and 431.2 arose from the C_18:3_ neutral loss as ketene from the each precursor ion. The other ions, m/z 671.5, 693.4, 695.5, and 697.5 also produced the similar MS^2^ spectra as described above, and were confirmed as phosphatidic acid.

Next we investigated whether RCNMV infection affects the amount of PA in plant leaves. *N*. *benthamiana* leaves were inoculated with RCNMV via agroinfiltration. At 2 dai, lipids were extracted and the amount of PA was analyzed by thin layer chromatography (TLC). Compared with control plant leaves infiltrated with *Agrobacterium* harboring an empty vector, the signal intensity of a lipid spot that showed a migration similar to that of soy-PA was increased in RCNMV-infected plant leaves (about 3-fold higher) (Fig 8A and 8B). To identify the lipid species of the spot, the same samples were again subjected to TLC, and the spot was scratched out from Coomassie Brilliant Blue R-250 stained TLC plates and subjected to LC/MS analysis. The lipid was identified as PA (Fig 8C–8E). We concluded that RCNMV infection upregulated PA accumulation in plants. It is known that expressions of *PLD* genes were induced by pathogen infection [50]. Therefore, the enhanced accumulation of PA in RCNMV-infected plants could be due to elevated accumulation of PLD through induction of *PLD* gene expression. To examine this possibility, we investigated whether mRNA levels of *NbPLDα* and *NbPLDβ* were upregulated in RCNMV-infected plants by RT-qPCR analysis. The accumulation levels of *NbPLDα* and *NbPLDβ* transcripts in RCNMV-infected plants were about 1.2- and 1.9-fold higher, respectively, than those in control plants, although the increase in *NbPLDα* transcripts was insignificant by the Student’s *t*-test (S9 Fig).

### *NbPLDα*- or *NbPLDβ*-knockdown plants exhibit reduced PA accumulation

We compared the accumulation of endogenous PA in *NbPLDα* or *NbPLDβ* knockdown plants with that in TRV control plants by TLC analysis. As expected from their predicted function, *NbPLDα*- or *NbPLDβ*-knockdown plants showed reduced accumulation of PA compared with TRV control plants (S10 Fig), suggesting that these PLDs contribute to PA production in *N*. *benthamiana*. These results further supported the idea that RCNMV-induced PLDs-derived PA plays an important role in RCNMV replication.

## Discussion

A growing number of studies have suggested that PLD and PLD-derived PA play vital roles in environmental responses in plants [19–22,50]. The properties of PA (i.e., normally present in small amounts, and rapidly and transiently accumulates in response to various environmental cues) seem to be suitable for its function in biotic and abiotic responses in which plants need to rapidly accommodate their surrounding environments. Indeed, PA has been shown to accumulate in response to several MAMPs and pathogen effector proteins, or SA that is a key hormone involved in plant resistance against biotrophic pathogens [23–29]. PA induces *PR* gene expression and cell death [28,33,34], and has been proposed to act as an important component in resistance to biotrophic pathogens such as *tobacco mosaic virus* and *Phytophthora parasitica*. Plants have diverse numbers of PLD isoforms and they appear to have distinct but somewhat overlapping functions in cellular responses [50]. In *Arabidopsis*, it is proposed that multiple PLD isoforms cooperatively contribute to AvrRpm1-triggered resistance [30]. In rice, PLDβ1 acts like a negative regulator of defense signaling because *PLDβ1*-knockdown rice plants exhibit constitutive ROS production, expression of *PR* genes, and enhanced resistance against pathogens [35]. In this study, we showed that PLD and PA are essential for and play a key role in RCNMV replication. Poor infection of RCNMV to *NbPLDα*- or *NbPLDβ*-knockdown plants was not due to constitutively activated defense responses, indicating that both NbPLDα and NbPLDβ act as essential host factors in RCNMV replication. The findings reveal novel aspects of PLD and PA in their roles during biotic stress responses in plants.

The replication of *Tomato bushy stunt virus* is enhanced by deletion of the *PAH1* gene encoding a PA phosphatase, which converts PA into DAG in yeast [51]. Moreover, ectopic expression of *Arabidopsis* PA phosphatase, Pah2 in *N*. *benthamiana* results in the inhibition of tombusviruses and RCNMV infection [51]. These findings suggest that PA is positively involved in the life cycles of these viruses. However, whether viruses manipulate PA production for viral replication has been unknown. In the current study, we showed that RCNMV replication proteins interacted with PA-producing enzymes, NbPLDα and NbPLDβ. RCNMV infection induced a high accumulation of PA in plant tissues, suggesting that RCNMV alters cellular lipid metabolism to establish a suitable environment for viral replication. It is known that transcription of *PLD* genes is induced by pathogen infection [50]. RCNMV infection increased the accumulation of *NbPLDα* and *NbPLDβ* transcripts (S9 Fig). Currently, whether the enhancement of PA accumulation observed in RCNMV-infected plants is due to the upregulation of *PLD* gene expression remains unknown. PLDs may be activated through direct or indirect interaction with RCNMV replication proteins. Although we failed to show the interaction between p88^pol^ and NbPLDα or NbPLDβ *in vivo* because p88^pol^ accumulates below detection limits in the absence of viral RNA replication in *N*. *benthamiana* [12], p88^pol^ interacted with PLDs, at least with NbPLDα in a co-IP assay in BYL. The interaction of p88^pol^ with PLDs seems to make sense for viral replication strategy because it brings them to the sites of replication. This strategy could increase PA only at VRCs and not at other cellular membranes where PA might affect cellular metabolism or activate the SA-mediated defense responses that is detrimental for successful viral infection. This is critical for the viral life cycle because PA interacts with p27 auxiliary replication protein (Fig 7) and enhances viral replication through upregulating the activity and/or assembly of the 480-kDa replicase complex (Fig 6). To our knowledge, this is the first demonstration of a functional role for PA in (+)RNA virus replication. It is likely that PA is involved in RNA replication of many (+)RNA viruses. Indeed, the replication proteins, 1a and 2a^pol^ of BMV, a virus unrelated to RCNMV, also interacted with NbPLDβ, and BMV RNA replication was sensitive to *n*-butanol, which is an inhibitor of PLDs-derived PA production (Fig 5). However, exogenously added PA did not affect the accumulation of BMV RNA (Fig 5), implying that the threshold of PA requirement for BMV RNA replication seems to be lower than that for RCNMV. The differential PA requirement may be explained by very weak affinity of BMV replication proteins with NbPLDα, which is more active in PA production than NbPLDβ (S10 Fig). Moreover, Dengue virus induces the accumulation of several lipids in infected mosquito cells, including PA [52]. It has been reported that Coxsackievirus B3 and mouse hepatitis coronavirus replication is insensitive to *n*-butanol [53,54]. However, because PA can also be formed through the combined action of phospholipase C and DAG kinase [55], it is unknown whether replication of these viruses depends on PA or not.

How does PA affect viral RNA replication? PA binding to proteins modulates the catalytic activity of target proteins, tethers proteins to the membranes, and promotes the formation and/or stability of protein complexes [55]. We found that exogenous PA enhanced the accumulation of newly synthesized viral RNA and the formation of 480-kDa replicase complexes in BYL *in vitro* translation/replication systems, and that p27 had affinity for PA *in vitro* (Figs 6 and 7). Therefore, PA could promote viral replicase activity and/or assembly directly. The 480-kDa replicase complexes contain p27 oligomer. Therefore, it is possible that PA-binding to p27 in the replicase complexes assists the conformational change of p27 that is suitable for RNA synthesis. Alternatively, PA could serve as an assembly platform for host PA-binding proteins. PA binds to various proteins, including transcription factors, kinases, phosphatases, enzymes involved in central metabolism, and proteins involved in vesicular trafficking and cytoskeletal rearrangements [20,21]. Several known cellular PA-binding proteins were also identified in our LC/MS/MS analysis (S1 Table). These include NADPH oxidase [56], glyceraldehyde-3-phosphate dehydrogenase (GAPDH) [57,58], and SNF1-related kinase [58]. RCNMV-induced accumulation of high PA levels may facilitate the recruitment of these PA-binding proteins to viral replication sites. One of the candidate proteins is GAPDH-A. Although GAPDH-A is not required for RCNMV replication, it recruits RCNMV movement protein to viral replication sites and plays an important role in virus cell-to-cell movement [59]. Therefore, PA may also function in bridging viral replication and cell-to-cell movement. RCNMV induces large ER aggregates in infected cells, which are thought to be viral RNA replication factories [13–16]. PA may play a direct role in the formation of RNA replication factories. In support of this hypothesis, deletion of *pah1* in yeast causes the expanded ER membranes and leads to enhanced TBSV replication on these membranes, although TBSV replicates normally at peroxisomes [51]. In addition, it is also possible that PA might affect unknown cellular factors that are involved in viral RNA replication. Further studies are needed to elucidate the molecular functions of PA in viral RNA replication.

Our results suggest that RNA1 and RNA2 have differential PA requirements in RNA replication. How does this difference contribute to RCNMV infection? RNA1 requires a larger amount of PA for maximum efficiency of its replication than that required by RNA2. This may reflect differences in the translation and replication mechanisms between RNA1 and RNA2. Translation of MP from RNA2 couples with RNA replication: only progeny RNA2 generated *de novo* through the RNA replication pathway could function as mRNA [60]. Poor enhancement of RNA2 replication by PA may be beneficial for switching from replication to translation. By contrast, because RNA1 has a cap-independent translation enhancer that is an effective recruiter of translation factors [61], newly synthesized RNA1 serves as a template for further translation of the replication proteins that would activate PA production in infected cells. Moreover, RNA1 also serves as a template for transcription of subgenomic RNA, which encodes CP. Therefore, PA-mediated enhancement of RNA1 replication may be suitable for the production of CP subgenomic RNA during the late stage of viral infection. High PA requirement for maximum RNA1 replication may explain the use of both NbPLDα and NbPLDβ as essential host factors.

A growing number of studies have suggested that multiple lipid species affect virus replication and that (+)RNA viruses employ a multifaceted strategy to rewire host machinery involved in lipid transport and synthesis [62]. HCV infection stimulates the production of cellular PC, PE, and PI4P and HCV co-opts PI4KIIIα for replication [63,64]. In addition, HCV infection stimulates the accumulation of cellular sphingomyelin, which binds to and activates HCV NS5B polymerase [65,66]. Enterovirus utilizes PI4KIIIβ for RNA replication and viral RdRP 3D^pol^ binds to PI4P [67]. Enteroviruses also upregulate cellular uptake of fatty acids, which are channeled toward highly upregulated PC synthesis in infected cells [68]. The elevated PC has been proposed to serve as a building block for the formations of the viral replication factory [68, 69]. Recently, it has been shown that PE and PC stimulate TBSV RdRP activity *in vitro* [70]. RCNMV p27 showed affinity, not only for PA but also for other phospholipids such as PI4P *in vitro*. However, our LC/MS/MS analysis failed to detect any PI4P-producing enzymes, such as PI4K. Combined lipidomics, proteomics, and transcriptome analysis will be helpful for a comprehensive understanding of lipid species involved in viral RNA replication.

## Materials and Methods

### Gene cloning and plasmid construction

Plasmids given the prefix ‘‘pBIC” were used for *Agrobacterium* infiltration, ‘‘pUC”, ‘‘pRC” and ‘‘pR” were used for *in vitro* transcription, ‘‘pCold” was used for protein expression in *Escherichia coli*. pUCR1 [71] and pRC2_G [72] are full-length cDNA clones of RNA1 and RNA2 of the RCNMV Australian strain, respectively. pB1TP3, pB2TP5, and pB3TP8 are full-length cDNA clones of RNA1, RNA2, and RNA3 of the BMV M1 strain, respectively [73] (generous gift from Paul Ahlquist). The constructs described previously used in this study include pBICp27 [71], pBICp88 [71], pBICR2 [71], pBICR1R2 [71], pBICp19 [71], pCOLDIp27-FLAG [49], pCOLDIp27N-FLAG [49], and pCOLDIp27C-FLAG [49]. pUC118 was purchased from Takara Bio Inc. (Shiga, Japan). *Escherichia coli* DH5α was used for the construction of all plasmids. All plasmids constructed in this study were verified by sequencing.

RNA extraction from *Nicotiana benthamiana* leaves was performed using an RNeasy Plant Mini Kit (Qiagen, Hilden, Germany). Reverse-transcription-PCR (RT-PCR) was catalyzed by Superscript III reverse transcriptase (Invitrogen) using oligo (dT) [16]. Primers to amplify coding sequences of *NbPLDα* or *NbPLDβ* were designed based on the *N*. *benthamiana* RNA seq data (Transcriptome version 5: http://sydney.edu.au/science/molecular_bioscience/sites/benthamiana/) [74].

#### pBICp27-His-FLAG

A PCR fragment from pBICp27 was amplified using primers 5'-CGGGGATCCATGGGTTTTATAAATCTTTCG-3' and 5'-CGGGGATCCCTACTTGTCATCGTCGTCCTTGTAATCATGATGATGATGATGATGAAAATCCTCAAGGGATTTG-3'. The amplified fragment was digested with BamHI and inserted into the corresponding region of pBICP35.

#### pBICp88-His-FLAG

A PCR fragment from pBICp88 was amplified using primers 5'-CCGGGTACCATGGGTTTTATAAATCTTTCG-3' and 5'- CGGGGTACCTTACTTGTCATCGTCGTCCTTGTAATCATGATGATGATGATGATGTCGGGCTTTGATTAGATCTTTG-3'. The amplified fragment was digested with KpnI and inserted into the corresponding region of pBICP35.

#### pBYLp27-HA

A PCR fragment from pUBp27-HA [16] was amplified using primers 5'- CTGGCGCGCCATGGGTTTTATAAATCTTTCGCTTTTTGATGTGG-3' and 5'-CTGGCGCGCCTTAAGCGTAATCTGGAACATCGTATGGGTA-3'. The amplified fragment was digested with AscI and inserted into the corresponding region of pBYL2 [12].

#### pBYLp88-HA

A PCR fragment from pUBp88-HA [16] was amplified using primers 5'- CTGGCGCGCCATGGGTTTTATAAATCTTTCGCTTTTTGATGTGG-3' and 5'-CTGGCGCGCCTTAAGCGTAATCTGGAACATCGTATGGGTA-3'. The amplified fragment was digested with AscI and inserted into the corresponding region of pBYL2.

#### pBYLNbPLDα-FLAG

A PCR fragment from cDNA derived from *N*. *benthamiana* was amplified using primers 5'-CTGGCGCGCCATGGCTCAGATTCTGCTTCATGGAACTCTCC-3' and 5'-CTGGCGCGCCCTACTTGTCATCGTCGTCCTTGTAATCTGTAGTGAGGATAGGAGGAAGG-3'. The amplified fragment was digested with AscI and inserted into the corresponding region of pBYL2.

#### pBYLSP6NbPLDβ-FLAG

A PCR fragment from cDNA derived from *N*. *benthamiana* was amplified using primers 5'-CTGGCGCGCCATGGCTCATTTGTCTTATTCTGATTCTATTAG-3' and 5'-CTGGCGCGCCTCACTTGTCATCGTCGTCCTTGTAATCGATATCTGGAAAGGTTTCACAGCCAGGGAG-3'. The amplified fragment was digested with AscI and inserted into the corresponding region of pBYL2 to generate pBYLNbPLDβ-FLAG. A PCR fragment from pBYLNbPLDβ-FLAG was amplified using primers 5'-GAGGATCCCCGATTTAGGTGACACTATAGAGACCCAAGCTGGCGCGCCATGGCTCATTTGT-3' and 5'-AGTCCCGGGCACACCCTTATACTCGTGATCTCCTCC-3'. The amplified fragment was digested with BamHI and XmaI, and inserted into the corresponding region of pBYLNbPLDβ-FLAG.

#### pBYLAtPAH1-HA

A PCR fragment from cDNA derived from *A*. *thaliana* was amplified using primers 5'-CTGGCGCGCCATGAGTTTGGTTGGAAGAGTTGGGAGTTTGATTT-3' and 5'-GCTGGCGCGCCTCAAGCGTAATCTGGAACATCGTATGGGTATTCAACCTCTTCTATTGGCAGTTTCC-3'. The amplified fragment was digested with AscI and inserted into the corresponding region of pBYL2.

#### pBYLFLuc-HA

A PCR fragment from pLUCpA60 [75] was amplified using primers 5'-CTGGCGCGCCATGGAAGACGCCAAAAACATAAAGAAAGGCCC-3' and 5'-CTGGCGCGCCTTAAGCGTAATCTGGAACATCGTATGGGTACACGGCGATCTTTCCGCCCTTCTTGGCC-3'. The amplified fragment was digested with AscI and inserted into the corresponding region of pBYL2.

#### pBYLFLuc-FLAG

A PCR fragment from pLUCpA60 [75] was amplified using primers 5'-CTGGCGCGCCATGGAAGACGCCAAAAACATAAAGAAAGGCCC-3' and 5'-CTGGCGCGCCTTACTTGTCATCGTCGTCCTTGTAATCCACGGCGATCTTTCCGCCCTTCTTGGCC-3'. The amplified fragment was digested with AscI and inserted into the corresponding region of pBYL2.

#### pBICp27-mCherry

A PCR fragment from pUBp27-mCherry [15] was amplified using primers 5'-AGGATCCGGATGGGTTTTATAAATCTTTCGCTTTTTGATGTGG-3' and 5'-GGGGTACCCTACTTGTACAGCTCGTCCATGCCGCCGGTGG-3'. The amplified fragment was digested with BamHI and KpnI, and inserted into the corresponding region of pBICP35 [76].

#### pBICsGFP-NbPLDα

A PCR fragment from pBYLNbPLDα-FLAG was amplified using primers 5'-CTGGCGCGCCATGGCTCAGATTCTGCTTCATGGAACTCTCC-3' and 5'-CTGGCGCGCCCTATGTAGTGAGGATAGGAGGAAGGTAGTCAG-3'. The amplified fragment was digested with AscI and inserted into the corresponding region of pBICsGFP [13].

#### pBICsGFP-NbPLDβ

A PCR fragment from pBYLNbPLDα-FLAG was amplified using primers 5'-CTGGCGCGCCATGGCTCATTTGTCTTATTCTGATTCTATTAG-3' and 5'-CTGGCGCGCCTCAGATATCTGGAAAGGTTTCACAGCCAGGGAG-3'. The amplified fragment was digested with AscI and inserted into the corresponding region of pBICsGFP.

#### pTVNbPLDα

A PLDα cDNA fragment was amplified from cDNA derived from *N*. *benthamiana* (NbPLDα) RNA using primers 5'-ATCCCCCGGGAGTTCCTTGTAGCTCTGTGACATCCCC-3' and 5'-GCAGCCCGGGTCAGCCAACATAAACCAGAGATCGATGGACGG-3'. The generated PCR product was then cloned in the antisense orientation into the XmaI site of pTV00 [77].

#### pTVNbPLDβ

A PLDβ cDNA fragment was amplified from cDNA derived from *N*. *benthamiana* (NbPLDβ) RNA using primers 5'-CCCCCGGGTCGACTTTTCTGACTGAGTTCCTGAGGTGTAGCAGG-3' and 5'-AGCCCGGGTTGATACCAATGGAGATTGCTCTAAAAATTGCC-3'. The generated PCR product was then cloned in the antisense orientation into the XmaI site of pTV00.

### Plant growth conditions

*N*. *benthamiana* plants were grown on commercial soil (Tsuchi-Taro, Sumirin-Nosan-Kogyo Co. Ltd.) at 25 ± 2°C and 16 h illumination per day.

### RNA preparation

RCNMV RNA1 and RNA2 were transcribed from SmaI-linearized pUCR1 and pRC2_G, respectively, using T7 RNA polymerase (TaKaRa Bio, Inc). BMV RNAs were transcribed from EcoRI-linearized pB plasmids using T7 RNA polymerase and capped with a ScriptCapm7G capping system (Epicentre Biotechnology). Capped mRNAs were transcribed from NotI-linearized pBYL plasmids using T7 or SP6 RNA polymerase (TaKaRa Bio, Inc) and capped with a ScriptCapm7G capping system (Epicentre Biotechnology). All transcripts were purified with a Sephadex G-50 fine column (Amersham Pharmacia Biotech). RNA concentration was determined spectrophotometrically, and its integrity was verified by agarose gel electrophoresis.

### Tandem affinity purification

Four-week-old *N*. *benthamiana* plants were agroinfiltrated as described previously [15]. At 2 days postinfiltration (dpi), total proteins were extracted from 6 g of leaves in 10 ml of buffer A (50 mM HEPES, 150 mM NaCl, 0.1% 2-mercaptoethanol, 0.5% Triton X-100, 5% glycerol, pH 7.5) containing 30 mM imidazole and a cocktail of protease inhibitors (Roche). Following the removal of cell debris by filtering the mixture through cheesecloth, the extract was centrifuged at 21,000*g* at 4°C for 10 min and the supernatant was mixed with Ni-NTA beads (400 μl) (Qiagen, Hilden, Germany) and incubated for 1 h at 4°C with gentle mixing. The beads were washed three times with 1 ml of buffer A containing 100 mM imidazole. The bound proteins were eluted with 1 ml of buffer A containing 500 mM imidazole. The eluted proteins were mixed with 50 μl of ANTI-FLAG M2-Agarose Affinity Gel (Sigma-Aldrich) and incubated for overnight at 4°C with gentle mixing. The beads were washed 3 times with 1 ml of buffer A. The bound proteins were eluted with 300 μl of buffer A containing 150 μg/ml 3 × FLAG peptides (Sigma-Aldrich). The eluted proteins were concentrated by acetone precipitation and dissolved in 1 × NuPAGE sample buffer (Invitrogen). The purified proteins were separated by sodium dodecyl sulfate (SDS)-PAGE (NuPAGE 3%–12% bis-Tris gel: Invitrogen) and visualized by silver staining (Wako, Osaka, Japan). Proteins in excised gel pieces were subjected to digestion with trypsin, LC–MS/MS analysis, and MASCOT searching as described previously [12].

### Silencing of *PLDα* and *PLDß* in *N*. *benthamiana*

Appropriate combinations of silencing vectors were expressed via *Agrobacterium* infiltration in 3- to 4-week-old *N*. *benthamiana* plants as described previously [16]. At 18 dpi, the leaves located above the infiltrated leaves were inoculated with *in vitro* transcribed RNA1 and RNA2 (500 ng each). At 2 days after inoculation, three inoculated leaves from three different plants infected with the same inoculum were pooled, and total RNA was extracted using RNA extraction reagent (Invitrogen), treated with RQ1 RNase-free DNase (Promega, Madison, WI), purified by phenol–chloroform and chloroform extractions, and precipitated with ethanol. Viral RNAs were detected by northern blotting, as described previously [16]. The mRNA levels of *NbPLDα* and *NbPLDß* were examined by RT-PCR using primer pairs 5′ -TATCAAGGTAGAGGAGATAGGTGC-3′ and 5′-TACATCATCTCCATCGTTCTCCTC-3′, and 5′-GAAGGCTTCAAAGCGCCATG-3′ and 5′-CTTAGGCAAGGGACATCAGC-3′, respectively. As a control to show the equal amounts of cDNA templates in each reaction mixture, the ribulose 1,5-biphosphate carboxylase small subunit gene (*RbcS*), a gene that is constitutively expressed, was amplified by RT-PCR as described previously [16].

### Protoplasts experiments

*N*. *benthamiana* protoplasts were prepared according to Kaido *et al*. (2014) [59]. *N*. *benthamiana* protoplasts were inoculated with RCNMV RNA1 (1.5 μg) and RNA2 (0.5 μg) and incubated with various concentrations of *n*-butanol (Sigma-Aldrich), *tert*-butanol (Sigma-Aldrich), phosphatidic acid (PA) (Soy-derived; Avanti Polar Lipid), phosphatidyl choline (PC) (Soy-derived; Avanti Polar Lipid) or phosphatidyl ethanolamine (PE) (Soy-derived; Avanti Polar Lipid) at 20°C for 18 h. Phospholipids were dissolved in dimethylsulfoxide. Total RNA was extracted and subjected to northern blotting, as described previously [15]. Each experiment was repeated at least three times using different batches of protoplasts.

### BYL experiments

The preparation of BYL was as described previously [38,46]. The BYL translation/replication assay was performed essentially as described previously [46]. Briefly, 300 ng of RNA1 was added to 30 μL of BYL translation/replication mixture in the presence of various concentrations of PA. The mixture was incubated at 17°C for 240 min. Aliquots of the reaction mixture were subjected to northern and immunoblotting analyses, as described previously [45,46,48].

### Coimmunopurification assay

Four-week-old *N*. *benthamiana* plants were agroinfiltrated as described previously [15]. At 4 days postinfiltration (dpi), total proteins were extracted from 0.33 g of leaves in 1 ml of buffer A containing a cocktail of protease inhibitors (Roche). Following the removal of cell debris by centrifugation at 21,000*g* at 4°C for 10 min, the supernatant was mixed with GFP-Trap agarose beads (10 μl) (ChromoTek) and incubated for 1 h at 4°C with gentle mixing. The beads were washed 3 times with 1 ml of buffer A. The bound proteins were eluted by addition of 1 × SDS gel loading buffer, followed by incubation for 3 min at 95°C. Protein samples were subjected to SDS-PAGE, followed by immunoblotting with appropriate antibodies.

FLAG- or HA-tagged proteins were expressed in BYL by adding an *in vitro* transcript. After incubation at 25°C for 120 min, a 10-μl bed volume of anti-HA Affinity Matrix (Roche) or ANTI-FLAG M2-Agarose Affinity Gel (Sigma-Aldrich) was added to the BYL and further incubated for 60 min with gentle mixing. The resin was washed three times with 200 μl of TR buffer [38] supplemented with 150 mM NaCl and 0.5% Triton X-100. The bound proteins were eluted by addition of 1 × SDS gel loading buffer, followed by incubation for 3 min at 95°C. Protein samples were subjected to SDS-PAGE, followed by immunoblotting with appropriate antibodies.

### Subcellular localization assays

Appropriate combinations of fluorescent protein-fused proteins were expressed in *N*. *benthamiana* leaves by *Agrobacterium* infiltration. Fluorescence of GFP and mCherry was visualized with confocal microscopy at 4 dai as described previously [59].

### Protein purification and lipid overlay assay

Protein expression in *E*. *coli* BL21 (DE3) and subsequent purification were done as described previously [15]. The concentration of purified protein was measured using a Coomassie Protein Assay Kit (Thermo Fisher Scientific, Waltham, MA). The purified protein was subjected to SDS-PAGE and visualized with Coomassie brilliant blue R-250 to check its purity.

Lipid overlay assays were conducted as recommended by the manufacture’s protocol. Briefly, the membrane (PIP Strips or Membrane Lipid Arrays; Echelon Bioscience Inc) was incubated in 3% fatty acid free BSA (Sigma-Aldrich) in a mixture of phosphate-buffered saline and 0.1% Tween 20 (PBST) for 1 h at room temperature (RT) and then incubated in the same solution containing 500 ng of purified recombinant protein for 1 h at RT. After washing three times with PBST, the membrane was incubated with a mouse anti-FLAG antibody (1:10000; Sigma-Aldrich) for 1 h at RT, followed by three washes with PBST. An anti-mouse IgG conjugated with horseradish peroxidase (1:10000; KPL) was used as a secondary antibody. Binding of proteins to phospholipids was visualized by incubation with a chemiluminescent substrate.

### Lipid extraction and TLC

Four-week-old *N*. *benthamiana* plants were inoculated with RCNMV via agroinfiltration. At 2 dai, 0.33 g of infiltrated leaves were ground in liquid nitrogen and extracted in 900 μl of water. Total lipids were extracted by adding 3 ml CHCl_3_/CH_3_OH (2:1, v/v) to each sample. The samples were vortexed and centrifuged at 1690*g*, at 4°C for 10 min. The organic phase was recovered and dried under nitrogen gas stream. Lipids were dissolved in 100 μl CHCl_3_/CH_3_OH (2:1, v/v). Subsequently, 5 μl of the samples were analyzed on TLC plates (Merck, Germany). The chromatography was performed using CHCl_3_/CH_3_OH/formic acid/acetic acid (12:6:0.6:0.4, v/v). Plates were air-dried, soaked in 10% CuSO4, and charred at 180°C for 10 min to visualize lipids.

To identify lipid species, the air-dried TLC plates were stained for an hour in a 0.03% Coomassie Brilliant Blue R-250 solution containing 20% of methanol and 0.5% of acetic acid. Destaining of the plates was performed with 20% methanol containing 0.5% acetic acid for 5 min. After drying the plates for a few minutes, the blue bands of interest were scratched out and transferred to glass tubes. The scratched silica gels were mixed with chloroform/methanol (3/7, v/v), followed by the centrifugation at 1,690*g*, at 4°C for 10 min. The supernatants were subjected to the LC-MS analysis using LCMS-IT-TOF mass spectrometer (Shimadzu, Kyoto, Japan). A TSK gel ODS-100Z column (2.0 × 150 mm, 5 μm, Tosoh, Tokyo, Japan) was eluted isocratically with acetonitrile/methanol/2-propanol/water (6/131/110/3, by volume) containing 19.6 mM of ammonium formate and 0.2% of formic acid at a flow rate of 0.2 mL/min. The MS was performed using an electrospray ionization interface operated in negative ion mode, under the following conditions: CDL temperature, 200°C; block heater temperature, 200°C; nebulizing gas (N_2_) flow, 1.5 L/min. The MS data were acquired in the range of m/z 600 to 1,000 using 10 msec ion accumulation time. The MS^2^ data were acquired in the range of m/z 125 to 500, using 50 msec ion accumulation time and automatic precursor ion selection in the range of m/z 650 to 750. CID parameters were follows: energy, 50%; collision gas (argon) 50%.

### Quantitative RT-PCR analysis

Total RNA extracted from *N*. *benthamina* leaves or protoplasts were subjected to reverse transcription using PrimeScript RT reagent Kit (Takara) using oligo-dT and random primers according to manufacturer’s protocol. Real-time PCR was carried out using SYBR Premix Ex Taq (RR420A, Takara). Primers used for real-time PCR analysis were listed in S2 Table. Quantitative analysis of each mRNA was performed using a Thermal cycler Dice Real Time System TP800 (Takara).

### Accession numbers

NbPLDα and NbPLDβ were registered through DDBJ and accession numbers LC033851 and LC033852, respectively, were given on March 11 2015.

## Supporting Information

S1 Table LC/MS/MS analysis of proteins copurified with the tagged RCNMV replication proteins. Each piece of silver-stained gel slices in Fig 1 was subjected to LC/MS/MS analysis.
(EPS)

S2 Table List of primers used in RT-qPCR analysis. (EPS)

S1 Fig Six-His- and FLAG-tagged p27 (p27-HF) and p88^pol^ (p88^pol^-HF) support RNA2 replication in *N. benthamiana*. Total RNA was extracted from *Agrobacterium*-infiltrated leaves expressing p27 plus p88 plus RNA2 or p27-HF plus p88-HF plus RNA2 at 2 days after infiltration, and the accumulations of positive- or negative-stranded RNA2 were analyzed by northern blotting. Ethidium bromide-stained rRNA was used as a loading control and is shown below the northern blots.
(TIFF)

S2 Fig The deduced amino acid sequences of NbPLDα (A) and NbPLDβ (B). Peptide sequences identified by LC/MS/MS analysis are highlighted in dark pink.
(TIFF)

S3 Fig p88^pol^ interacts with NbPLDα in a co-IP assay in BYL. Appropriate combinations of capped transcripts were added to BYL. After *in vitro* translation at 25°C for 2 hours, the extract was solubilized and subjected to immunoprecipitation of HA-tagged proteins by anti-HA antibody. Proteins were analyzed by immunoblot using antibodies as indicated.
(TIFF)

S4 Fig NbPLDα- or NbPLDβ-knockdown plants do not exhibit any symptoms. The tobacco rattle virus (TRV) vector harboring a partial fragment of *N*. *benthamiana PLDα* (TRV:NbPLDα) or *PLDβ* (TRV:NbPLDβ) was expressed in *N*. *benthamiana* by *Agrobacterium* infiltration. The empty TRV vector (TRV:00) was used as a control. Pictures were taken at 20 days after infiltration (dai). Note that the infiltrated plants show no morphological defects at this stage.
(TIFF)

S5 Fig Expression of defense-related and PLD genes in TRV:00, TRV:NbPLDα, or TRV:NbPLDβ infected *N. benthaminana*. The transcript accumulations of defense-related genes were analyzed by quantitative RT-PCR. TRV:NbPLDα or TRV:NbPLDβ was expressed in *N*. *benthamiana* by *Agrobacterium* infiltration. The empty TRV vector (TRV:00) was used as a control. Total RNA was extracted at 18 dai from the newly developed leaves. SA-signaling marker genes (*PR-1*, *PR-2*, and *PR-5*) (A), JA-signaling marker genes (*LOX1*, *PR-4*, and *PDF1*.*2*.) (B), ROS-detoxification enzymes (*APX*, *GST*, and *SOD*) (C), MAMP-triggered immunity (MTI) marker genes (*CYP71D20* and *ACRE132*) (D), mitogen-activated protein kinases (MAPKs) (*WIPK* and *SIPK*) (E), and PLDs (*PLDα* and *PLDβ*) (F). *UBQ3* was used as an internal control. Bars represent means and standard error of values obtained from two independent biological samples. Three technical replicates for each biological sample were examined. Replication of the experiment showed similar results.
(TIFF)

S6 Fig An inhibitor of PLDs impairs RCNMV RNA replication in tobacco BY-2 protoplasts. Protoplasts were inoculated with *in vitro* transcribed RNA1 and RNA2. The inoculated protoplasts were incubated at 20°C for 18 hours in the presence of *n*-butanol. Accumulation of RCNMV RNAs was analyzed by northern blotting. Ethidium bromide-stained ribosomal RNAs (rRNAs) are shown below the northern blots, as loading controls. sgRNA, subgenomic RNA; SR1f, a small RNA fragment that derived from non-coding region of RNA1 [47].
(TIFF)

S7 Fig Effects of *n*-butanol and *tert*-butanol on defense-related gene expressions in *N*. *benthamiana* protoplasts. The isolated protoplasts were incubated at 20°C for 18 hours in the presence of *n*-butanol (0.4% v/v), or *tert-*butanol (0.4% v/v). The transcript accumulations of defense-related genes were analyzed by quantitative RT-PCR. SA-signaling marker genes (*PR-1*, *PR-2*, and *PR-5*) (A), JA-signaling marker genes (*LOX1*, *PR-4*, and *PDF1*.*2*.) (B), ROS-detoxification enzymes (*APX*, *GST*, and *SOD*) (C), MAMP-triggered immunity marker (MTI) genes (*CYP71D20* and *ACRE132*) (D), and mitogen-activated protein kinases (MAPKs) (*WIPK* and *SIPK*) (E). *UBQ3* was used as an internal control. Bars represent means and standard error of values obtained from three independent biological samples. Three technical replicates for each biological sample were examined. Replication of the experiment showed similar results.
(TIFF)

S8 Fig An exogenously supplied phosphatidyl choline (PC) or phosphatidyl ethanolamine (PE) has no effects on RCNMV RNA replication. *N*. *benthamiana* protoplasts were inoculated with *in vitro* transcribed RNA1 and RNA2. The inoculated protoplasts were incubated at 20°C for 18 hours in the presence of phospholipids (0, 1, 2.5, or 5 μM). Accumulation of RCNMV RNA was analyzed by northern blotting. Ethidium bromide-stained RNAs (rRNA) is shown below the northern blots, as loading controls. The numbers below the images represent the relative accumulation levels (means ± standard error) of viral RNAs (RNA1 and RNA2, respectively) using the Image Gauge program, which were calculated based on three independent experiments. sgRNA, subgenomic RNA; SR1f, a small RNA fragment that derived from non-coding region of RNA1 [47].
(TIFF)

S9 Fig RCNMV upregulates mRNA levels of *NbPLDα* and *NbPLDβ*. The transcript accumulations of *PLD* genes were analyzed by quantitative RT-PCR. The accumulations of *NbPLDα* and *NbPLDβ* transcripts increased about 1.2- and 1.9-fold, respectively in RCNMV-infected plants compared with those in empty vector-expressing control plants. Asterisk indicates a significant (P<0.05; Student’s *t*-test) difference compared with the accumulation level of the transcript in the leaves from empty vector expressing *N*. *benthamiana*. *UBQ3* was used as an internal control. Bars represent means and standard error of values obtained from three independent biological samples. Three technical replicates for each biological sample were examined. Replication of the experiment showed similar results.
(TIFF)

S10 Fig NbPLDα- and NbPLDβ-knockdown *N. benthamiana* plants exhibit reduced accumulation of PA. TRV:NbPLDα or TRV:NbPLDβ was expressed in *N*. *benthamiana* by *Agrobacterium* infiltration. The empty TRV vector (TRV:00) was used as a control. Total lipids were extracted at 20 dai from the newly developed leaves. The extracted lipids were subjected to thin layer chromatography and phospholipids were visualized by CuSO_4_ staining. PA, phosphatidic acid.
(TIFF)
