# Supplementary figures and images for: Phosphatidic Acid Produced by Phospholipase D Promotes RNA Replication of a Plant RNA Virus
Source: PLoS Pathog. 2015 May 28;11(5):e1004909. doi: 10.1371/journal.ppat.1004909 (PMC4447390; doi:10.1371/journal.ppat.1004909)

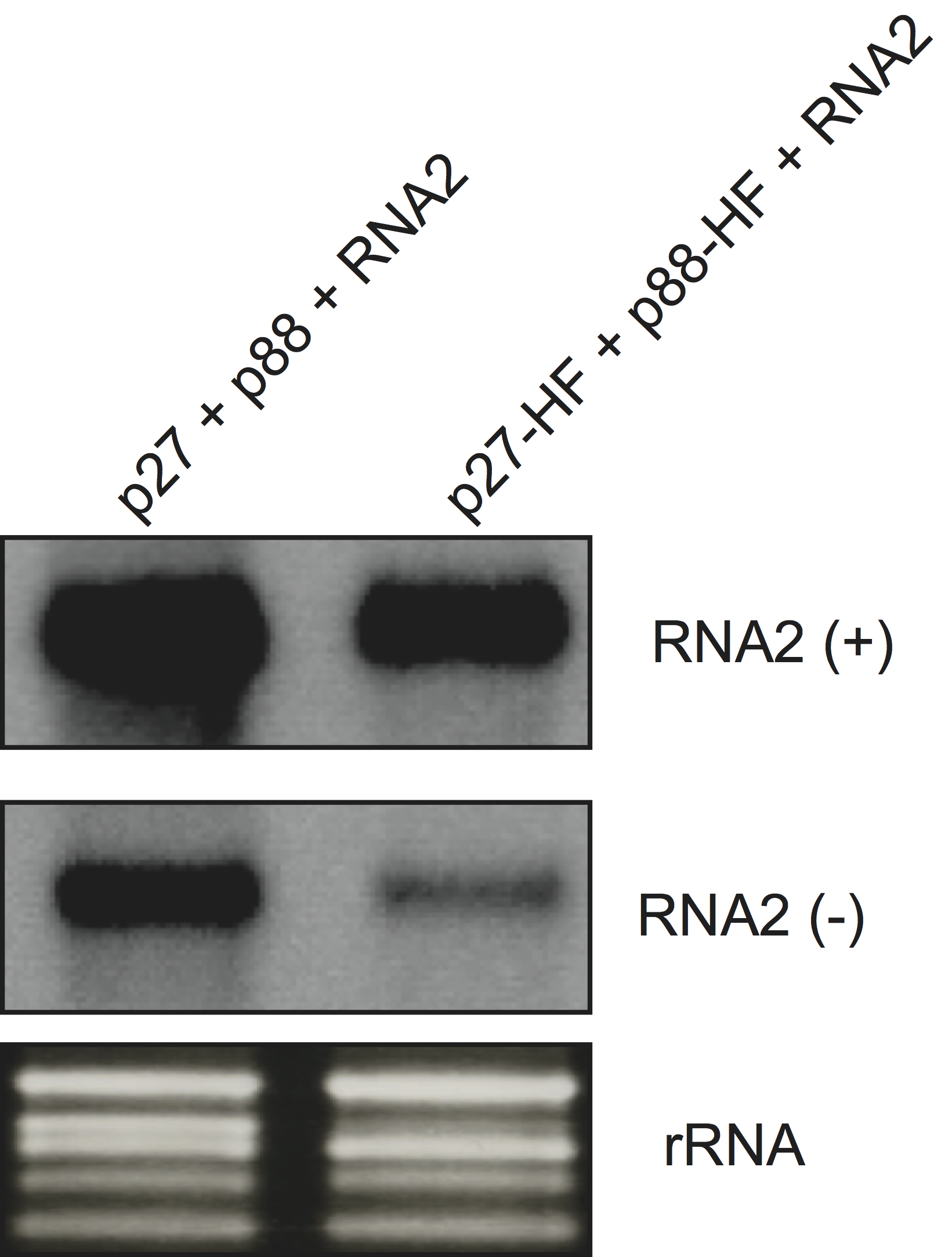

Supplement: S1 Fig — Total RNA was extracted from Agrobacterium-infiltrated leaves expressing p27 plus p88 plus RNA2 or p27-HF plus p88-HF plus RNA2 at 2 days after infiltration, and the accumulations of positive- or negative-stranded RNA2 were analyzed by northern blotting. Ethidium bromide-stained rRNA was used as a loading control and is shown below the northern blots. (TIFF) [file ppat.1004909.s003.tiff]

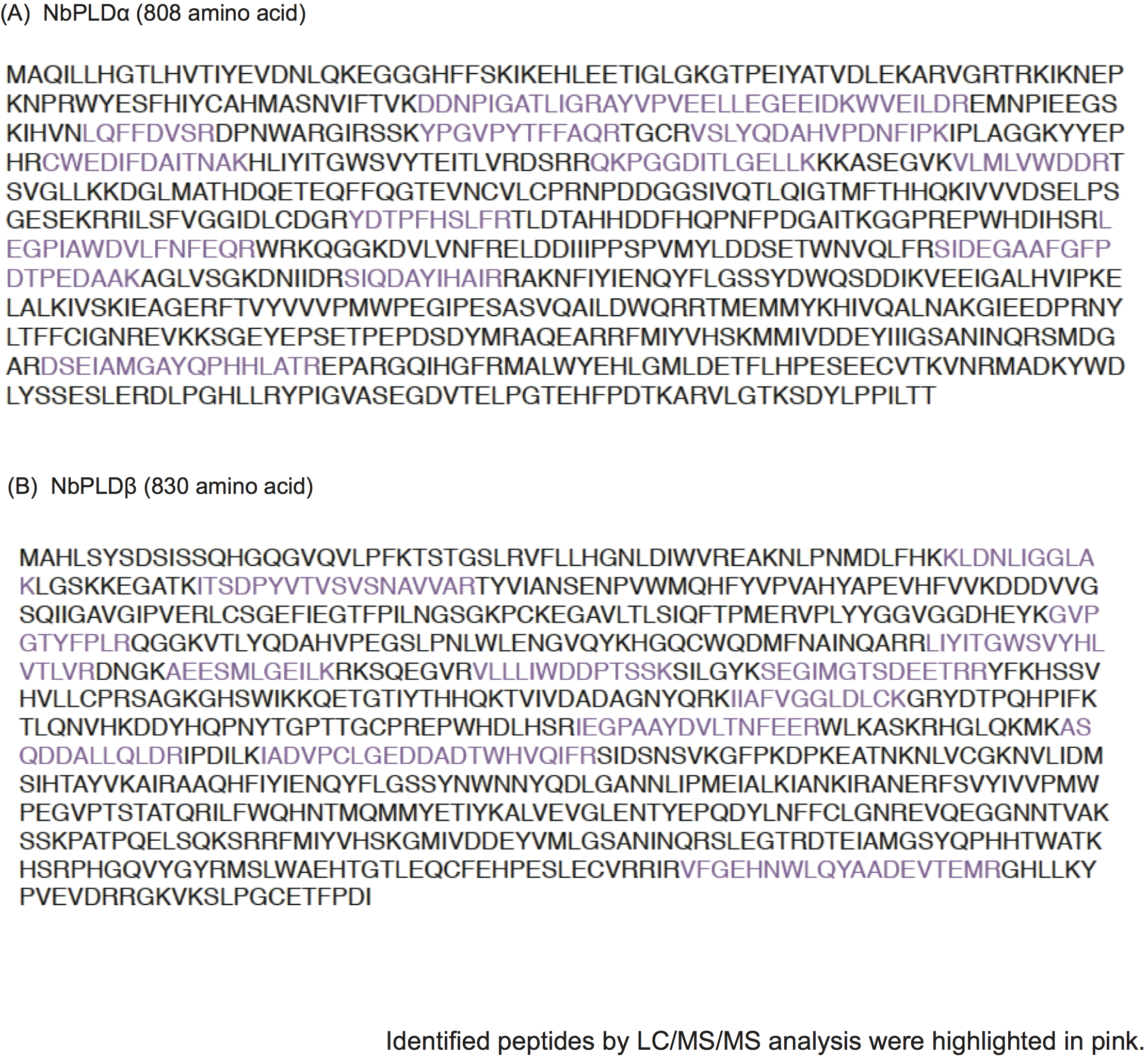

Supplement: S2 Fig — Peptide sequences identified by LC/MS/MS analysis are highlighted in dark pink. (TIFF) [file ppat.1004909.s004.tiff]

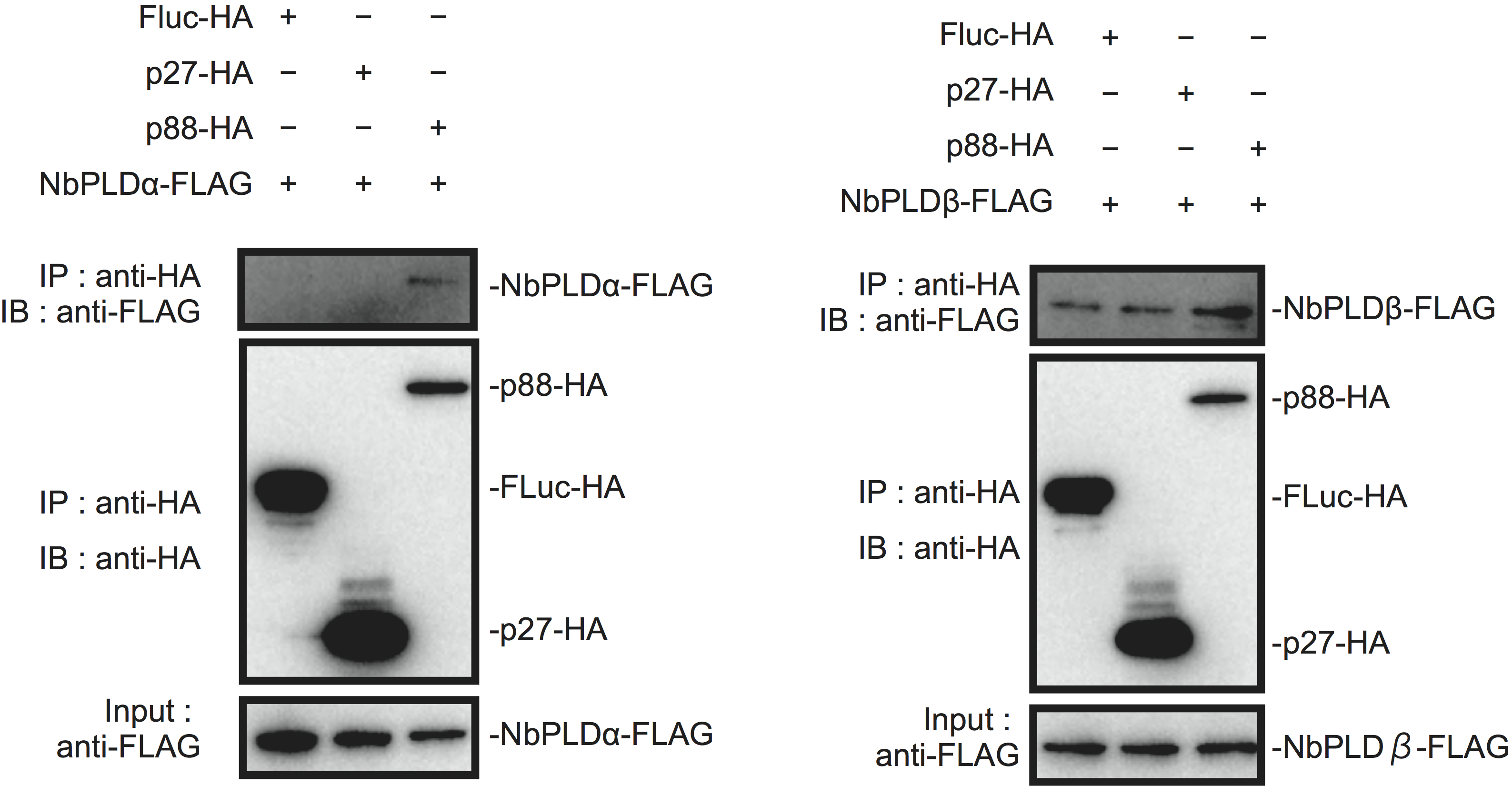

Supplement: S3 Fig — Appropriate combinations of capped transcripts were added to BYL. After in vitro translation at 25°C for 2 hours, the extract was solubilized and subjected to immunoprecipitation of HA-tagged proteins by anti-HA antibody. Proteins were analyzed by immunoblot using antibodies as indicated. (TIFF) [file ppat.1004909.s005.tiff]

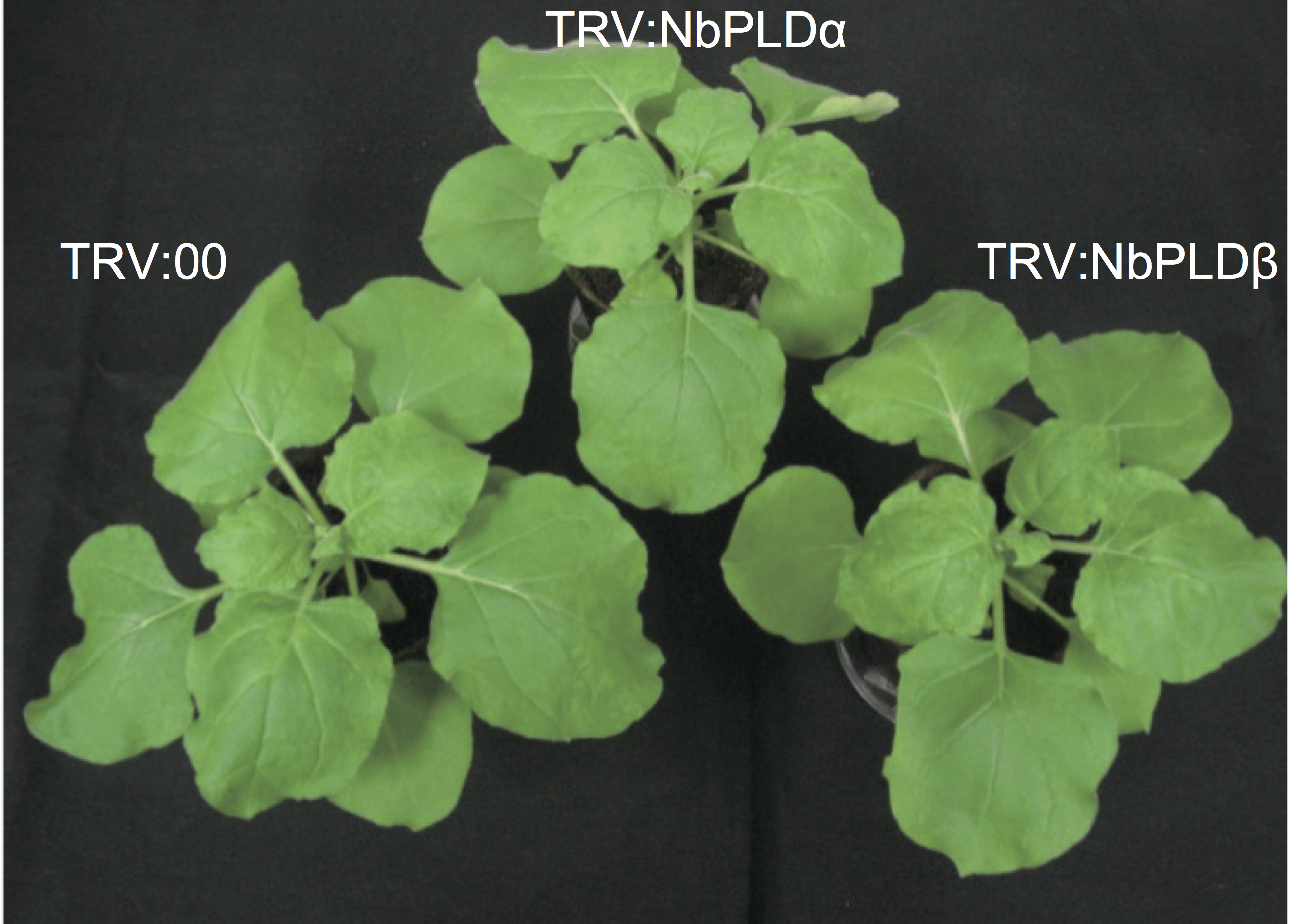

Supplement: S4 Fig — The tobacco rattle virus (TRV) vector harboring a partial fragment of N. benthamiana PLDα (TRV:NbPLDα) or PLDβ (TRV:NbPLDβ) was expressed in N. benthamiana by Agrobacterium infiltration. The empty TRV vector (TRV:00) was used as a control. Pictures were taken at 20 days after infiltration (dai). Note that the infiltrated plants show no morphological defects at this stage. (TIFF) [file ppat.1004909.s006.tiff]

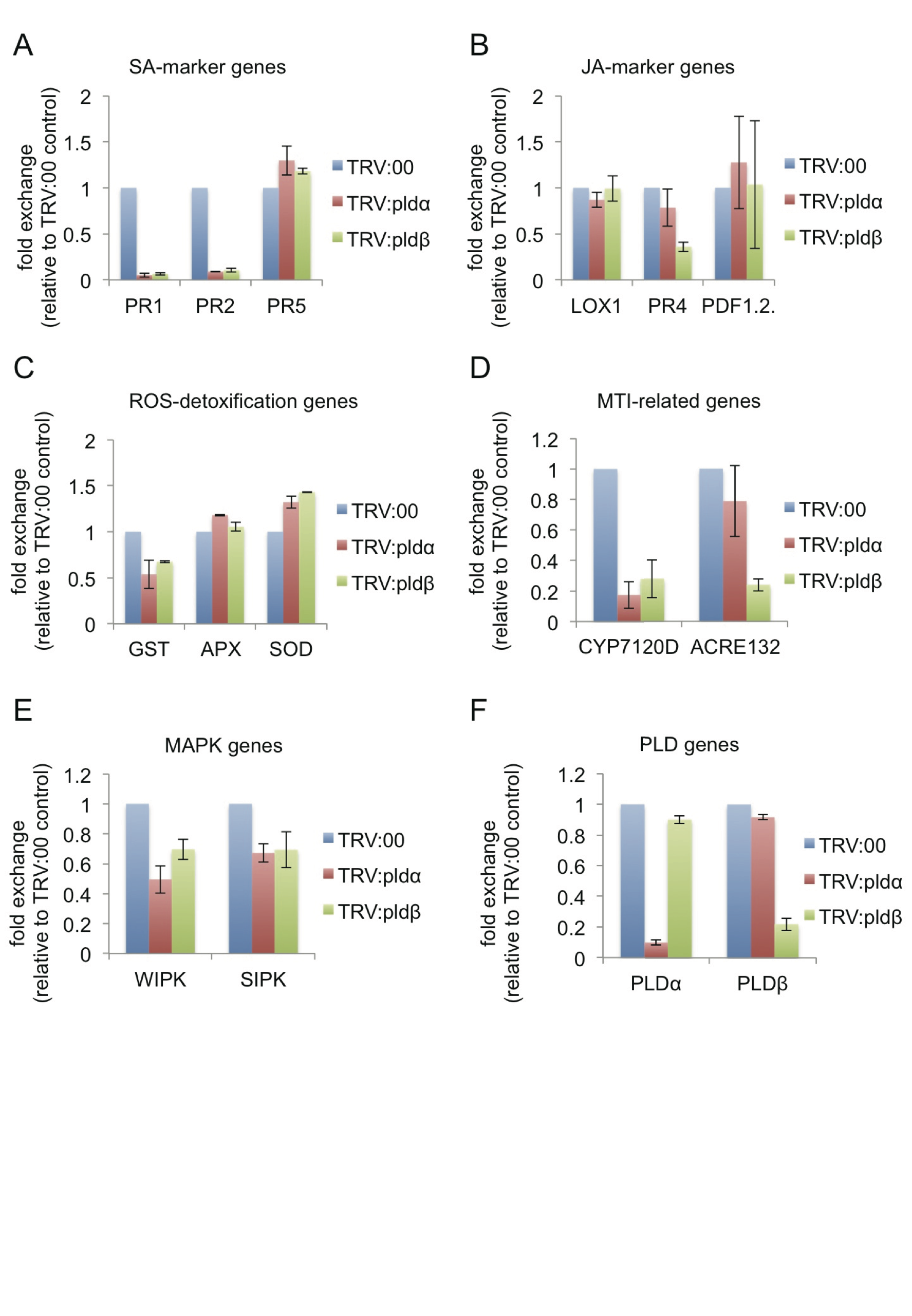

Supplement: S5 Fig — The transcript accumulations of defense-related genes were analyzed by quantitative RT-PCR. TRV:NbPLDα or TRV:NbPLDβ was expressed in N. benthamiana by Agrobacterium infiltration. The empty TRV vector (TRV:00) was used as a control. Total RNA was extracted at 18 dai from the newly developed leaves. SA-signaling marker genes (PR-1, PR-2, and PR-5) (A), JA-signaling marker genes (LOX1, PR-4, and PDF1.2.) (B), ROS-detoxification enzymes (APX, GST, and SOD) (C), MAMP-triggered immunity (MTI) marker genes (CYP71D20 and ACRE132) (D), mitogen-activated protein kinases (MAPKs) (WIPK and SIPK) (E), and PLDs (PLDα and PLDβ) (F). UBQ3 was used as an internal control. Bars represent means and standard error of values obtained from two independent biological samples. Three technical replicates for each biological sample were examined. Replication of the experiment showed similar results. (TIFF) [file ppat.1004909.s007.tiff]

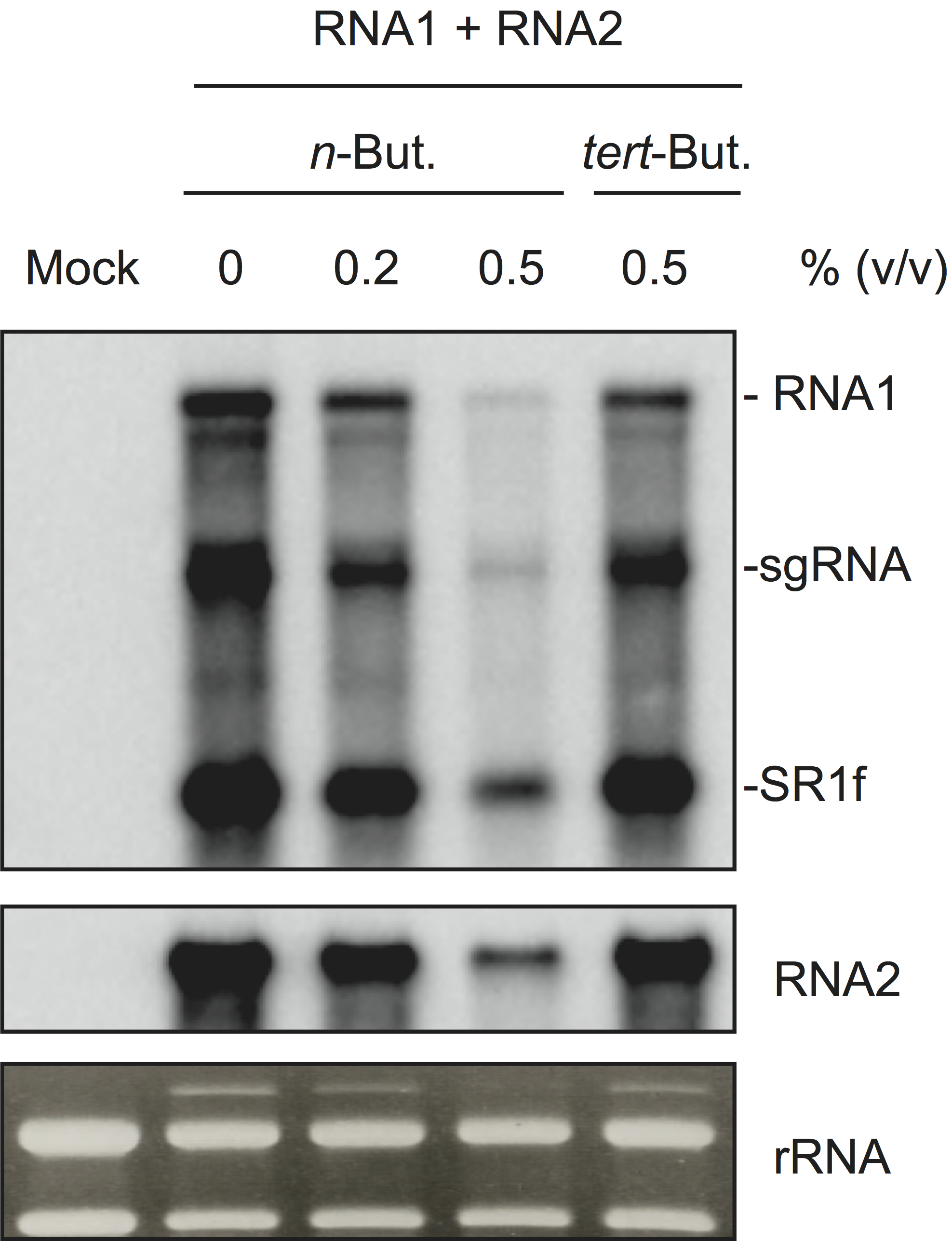

Supplement: S6 Fig — Protoplasts were inoculated with in vitro transcribed RNA1 and RNA2. The inoculated protoplasts were incubated at 20°C for 18 hours in the presence of n-butanol. Accumulation of RCNMV RNAs was analyzed by northern blotting. Ethidium bromide-stained ribosomal RNAs (rRNAs) are shown below the northern blots, as loading controls. sgRNA, subgenomic RNA; SR1f, a small RNA fragment that derived from non-coding region of RNA1 [47]. (TIFF) [file ppat.1004909.s008.tiff]

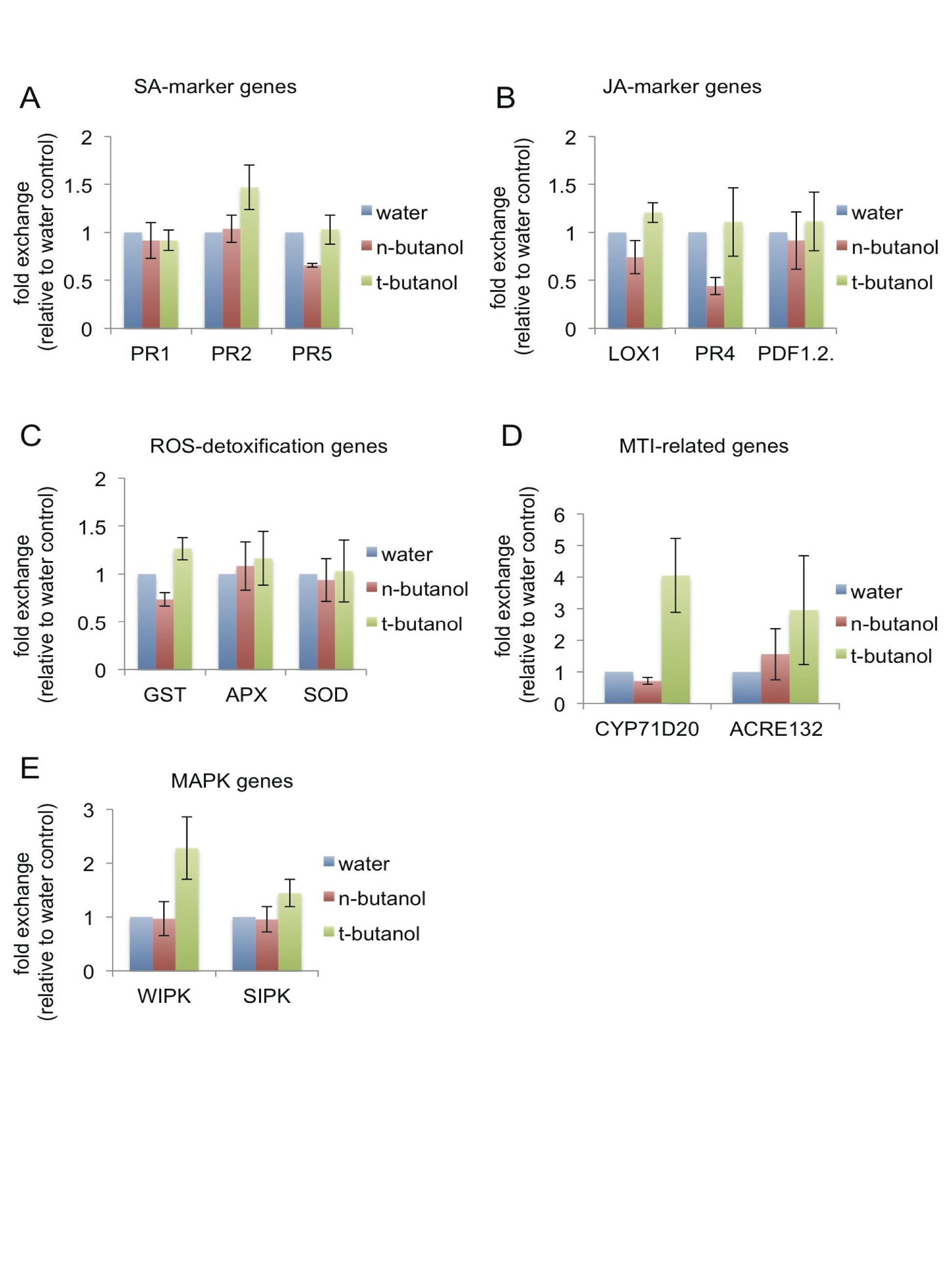

Supplement: S7 Fig — The isolated protoplasts were incubated at 20°C for 18 hours in the presence of n-butanol (0.4% v/v), or tert-butanol (0.4% v/v). The transcript accumulations of defense-related genes were analyzed by quantitative RT-PCR. SA-signaling marker genes (PR-1, PR-2, and PR-5) (A), JA-signaling marker genes (LOX1, PR-4, and PDF1.2.) (B), ROS-detoxification enzymes (APX, GST, and SOD) (C), MAMP-triggered immunity marker (MTI) genes (CYP71D20 and ACRE132) (D), and mitogen-activated protein kinases (MAPKs) (WIPK and SIPK) (E). UBQ3 was used as an internal control. Bars represent means and standard error of values obtained from three independent biological samples. Three technical replicates for each biological sample were examined. Replication of the experiment showed similar results. (TIFF) [file ppat.1004909.s009.tiff]

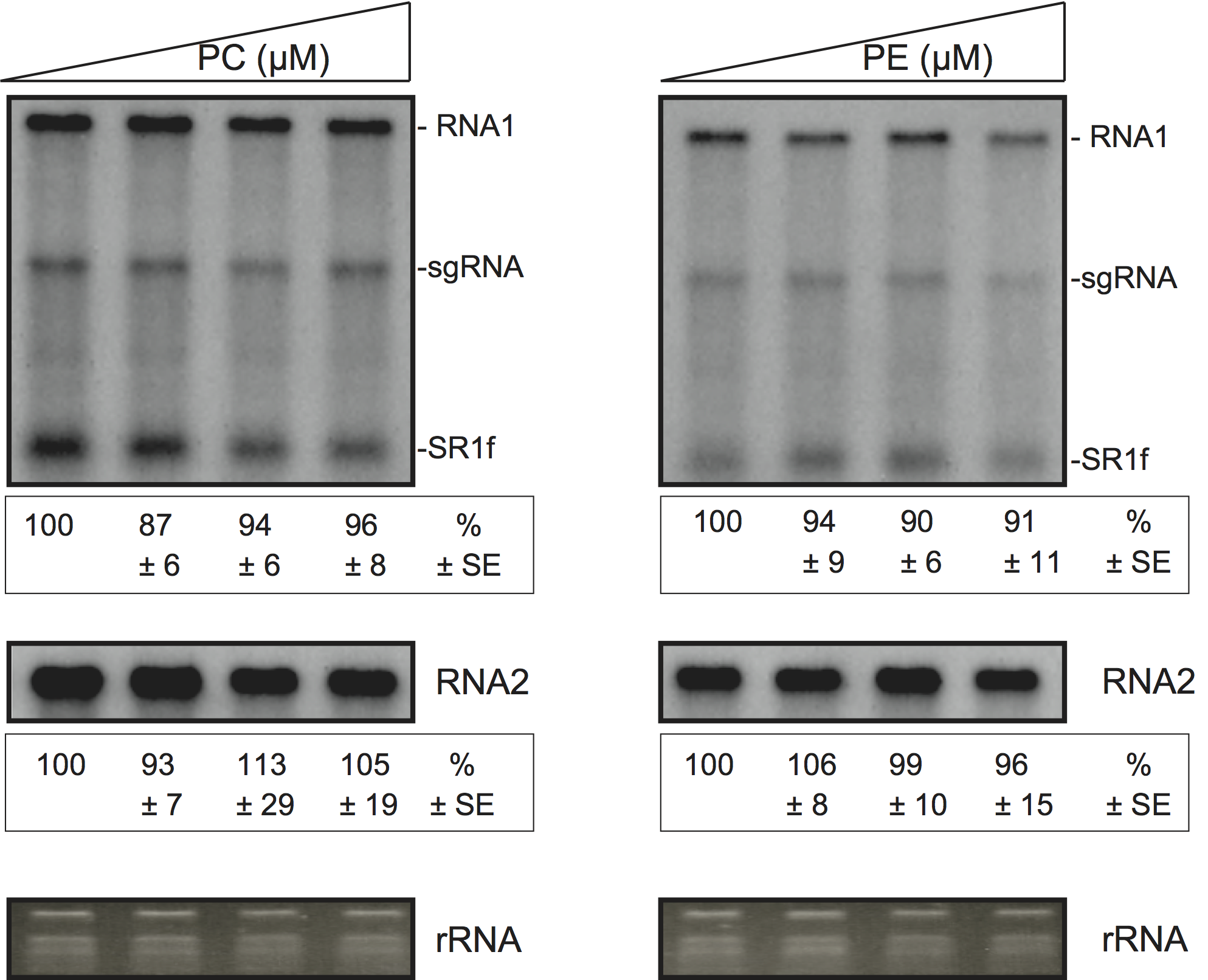

Supplement: S8 Fig — N. benthamiana protoplasts were inoculated with in vitro transcribed RNA1 and RNA2. The inoculated protoplasts were incubated at 20°C for 18 hours in the presence of phospholipids (0, 1, 2.5, or 5 μM). Accumulation of RCNMV RNA was analyzed by northern blotting. Ethidium bromide-stained RNAs (rRNA) is shown below the northern blots, as loading controls. The numbers below the images represent the relative accumulation levels (means ± standard error) of viral RNAs (RNA1 and RNA2, respectively) using the Image Gauge program, which were calculated based on three independent experiments. sgRNA, subgenomic RNA; SR1f, a small RNA fragment that derived from non-coding region of RNA1 [47]. (TIFF) [file ppat.1004909.s010.tiff]

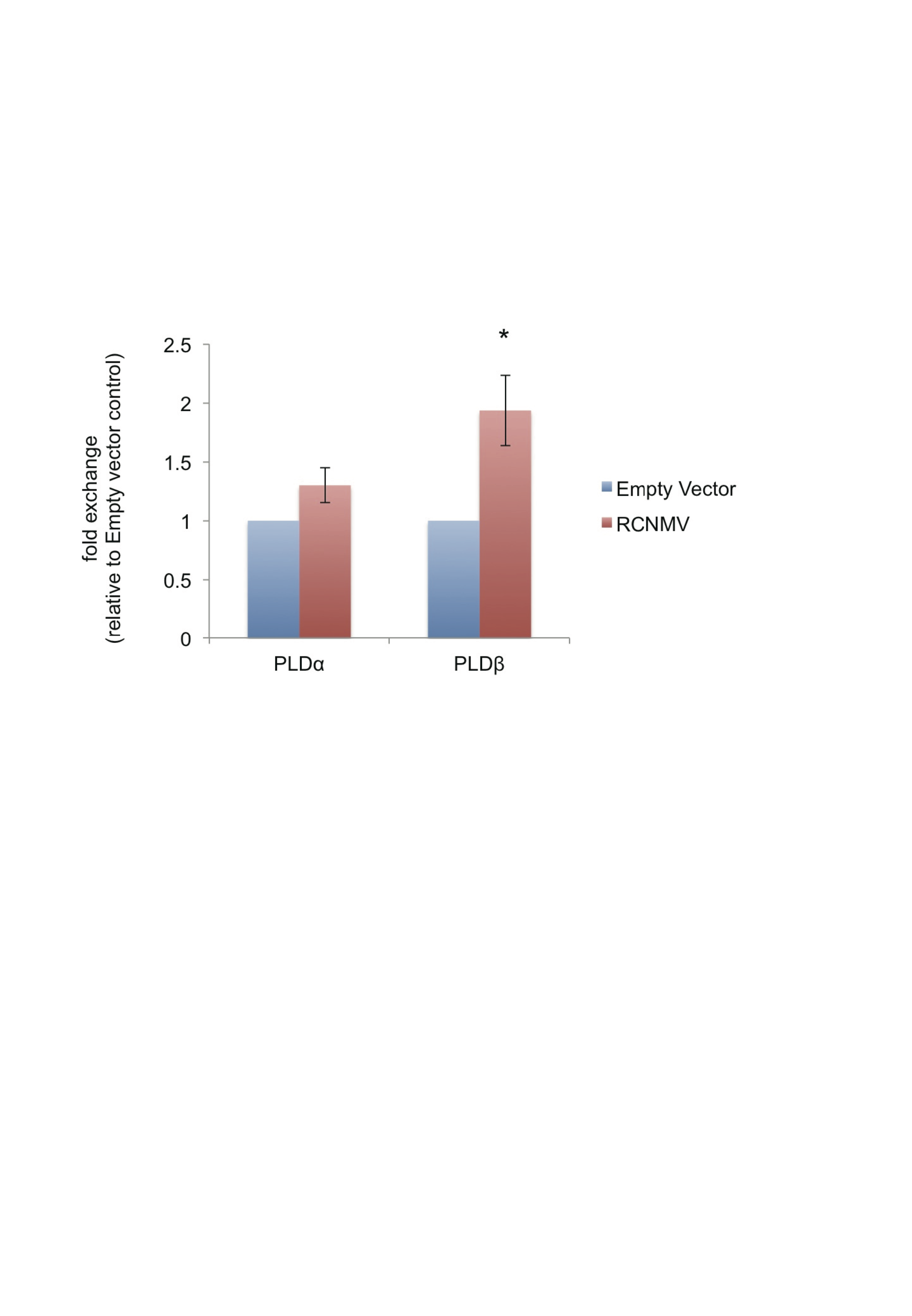

Supplement: S9 Fig — The transcript accumulations of PLD genes were analyzed by quantitative RT-PCR. The accumulations of NbPLDα and NbPLDβ transcripts increased about 1.2- and 1.9-fold, respectively in RCNMV-infected plants compared with those in empty vector-expressing control plants. Asterisk indicates a significant (P<0.05; Student’s t-test) difference compared with the accumulation level of the transcript in the leaves from empty vector expressing N. benthamiana. UBQ3 was used as an internal control. Bars represent means and standard error of values obtained from three independent biological samples. Three technical replicates for each biological sample were examined. Replication of the experiment showed similar results. (TIFF) [file ppat.1004909.s011.tiff]

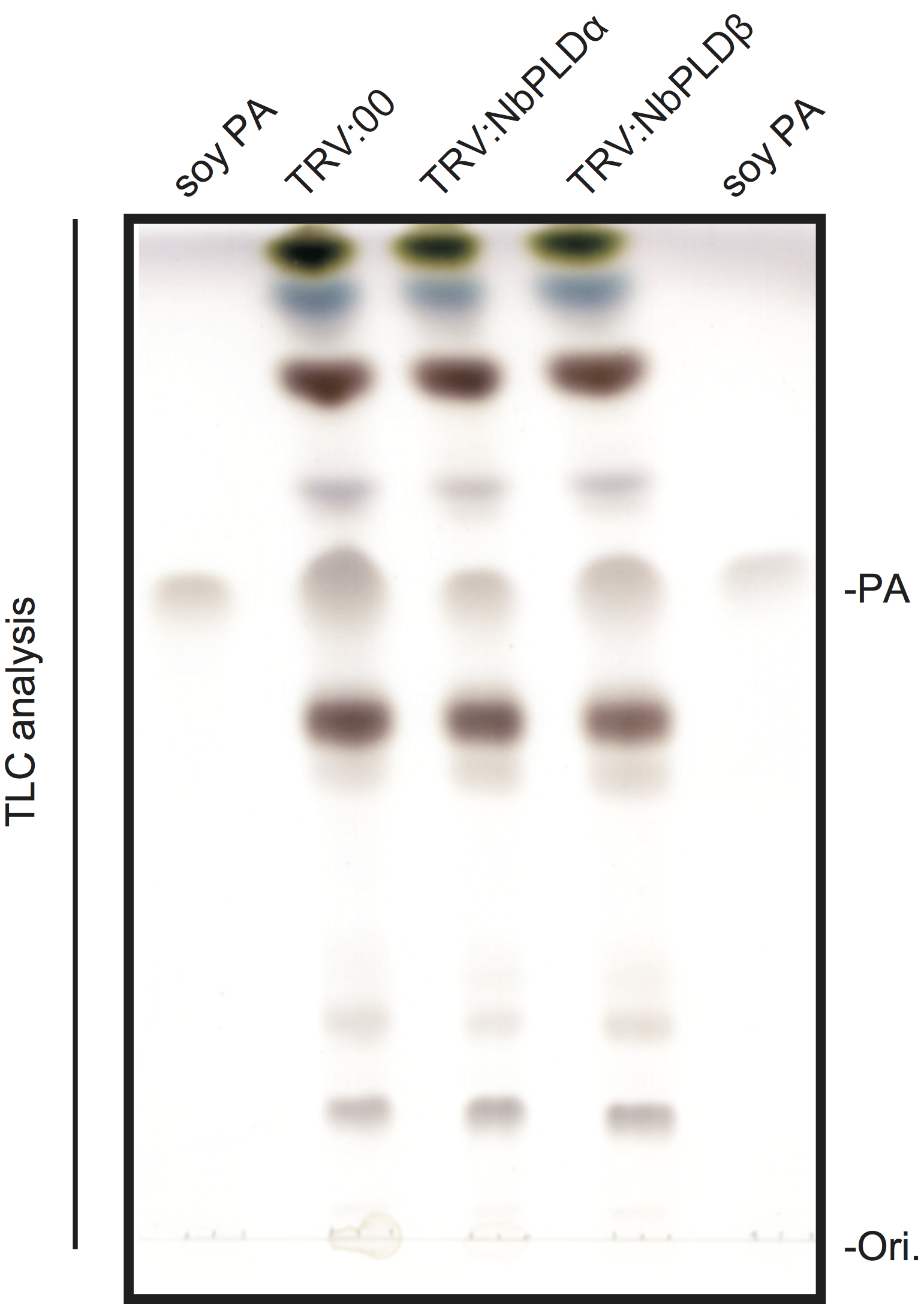

Supplement: S10 Fig — TRV:NbPLDα or TRV:NbPLDβ was expressed in N. benthamiana by Agrobacterium infiltration. The empty TRV vector (TRV:00) was used as a control. Total lipids were extracted at 20 dai from the newly developed leaves. The extracted lipids were subjected to thin layer chromatography and phospholipids were visualized by CuSO4 staining. PA, phosphatidic acid. (TIFF) [file ppat.1004909.s012.tiff]
